# Hexokinase 1 facilitates post‐germinative seedling growth through its catalytic function

**DOI:** 10.1111/tpj.70967

**Published:** 2026-07-08

**Authors:** Mengke Zhou, Ashwin Ganpudi, Anne C. Lincoln, Andrés Romanowski, Leon D. Matkovics, Karen J. Halliday

**Affiliations:** ^1^ Institute for Molecular Plant Sciences, School of Biological Sciences University of Edinburgh Edinburgh EH9 3BF UK; ^2^ Timing of Environmental Signalling, Laboratory of Molecular Biology, PSG Wageningen University & Research Wageningen 6708PB The Netherlands

**Keywords:** hexokinase, phytochrome, Skotomorphogenesis, hypocotyl, *Arabidopsis thaliana*

## Abstract

In darkness or dim light PHYTOCHROME INTERACTING FACTORS (PIFs) induce skotomorphogenic seedling growth, which is exemplified by elongated hypocotyls. Likewise, HEXOKINASE1 (HXK1) has been reported to promote hypocotyl growth under light and nutrient limiting conditions. HXK1 is known to operate as a glucose‐phosphorylating enzyme and as a glucose activated sensor‐signalling molecule. Earlier work implicated HXK1 sensor‐signalling in hypocotyl elongation; however, less is known of whether HXK1 enzymatic function and/or HXK1‐PIF pathway interaction are involved. We provide genetic evidence that HXK1‐mediated glucose‐phosphorylation is required for hypocotyl cell expansion in light limiting conditions. Application of glucose‐6‐phosphate, the HXK1 enzymatic product, restores short *gin2‐1/hxk1‐3* hypocotyls to wild‐type length. Further, components of nuclear‐located HXK1 sensor‐signalling complexes, comprising VHA‐B1 and RPT5B, or the Polycomb Repressive Complex 2 subunits SWN and CLF, do not contribute to this response. Unlike *gin2‐1/hxk1‐3*, the *vha‐B1, rpt5b swn‐7, clf28, clf29* alleles only disrupt hypocotyl growth following the application of exogenous glucose and not in control conditions. mRNA‐seq analysis illustrates that HXK1 and PIF signalling intersect at genes with known roles in light signalling. HXK1 imposes strong negative regulation on chloroplast and mitochondrial genomes, and also branched‐chain amino acid catabolism pathway genes, which can provide a source of respiratory substrates in starvation conditions. Our study establishes the importance of HXK1 enzymatic function in supporting hypocotyl cell expansion, amino acid metabolism and the transcriptional regulation of light signalling genes.

## Significance Statement

This study highlights the critical role of the HXK1‐G6P pathway in supporting post‐germinative hypocotyl cell expansion. HXK1 exerts regulatory control over the chloroplast and mitochondrial genomes and modulates the availability of respiratory substrates to synchronise cellular metabolism with carbon resource availability.

## INTRODUCTION

The first days of life are critical for plant survival; successful seedling establishment relies on the mobilisation of seed reserves and the perception of light through wavelength‐specific photoreceptors. These factors enable seedling emergence and the switch to photo‐autotrophic growth. HEXOKINASE 1 (HXK1), a metabolic enzyme with glucose sensor‐signalling properties, and PHYTOCHROME‐INTERACTING FACTORS (PIFs), play pivotal roles in coupling carbon availability to growth in the developing seedling (Krahmer et al., 2021; Lilley et al., 2012; Moore et al., 2003; Mu et al., 2023; Sairanen et al., 2012; Stewart et al., 2011).

In Arabidopsis and other oilseed plants, seed carbon resources are stored primarily in the form of triacylglycerols (TAGs) and seed storage proteins, which account for approximately 30–40% of the seed's dry weight each (Baud et al., 2008). After germination TAGs are metabolised and converted to sucrose. TAG‐derived fatty acids undergo β‐oxidation, forming acetyl‐CoA, which fuels the production of sucrose via the glyoxylate cycle and gluconeogenesis. Sucrose is subsequently transported throughout the seedling to support development (Eastmond et al., 2000; Yang & Benning, 2018). In sink tissues, sucrose is cleaved by either SUCROSE SYNTHASE to produce UDP‐glucose and fructose, or by INVERTASE to liberate glucose and fructose (Coculo & Lionetti, 2022). A vital step in the metabolism of these hexose sugars is their phosphorylation, which is mediated by HXK and FRUCTOKINASE (FRK). HXK is capable of phosphorylating both glucose and fructose, whereas FRK predominantly targets fructose. However, FRK shows a greater binding affinity for fructose compared to HXK, indicating the principal role of HXK is the phosphorylation of glucose to glucose‐6‐phosphate (G6P) (Claeyssen & Rivoal, 2007; Granot, 2007; Harrington & Bush, 2003). In Arabidopsis, there are six different members of the HXK family: HXK1–3, and HEXOKINASE‐LIKE (HKL) 1–3 (Karve et al., 2008). A primary distinction between HXK and the HKL proteins is that the latter lack glucokinase activity, likely due to an insertion or deletion at the adenosine binding site. Across the family, the most well‐studied member is HXK1.

Beyond its enzymatic function, HXK1 also serves a signalling function through molecular interactions. HXK1 binding to KINγ, a regulatory subunit of SNF1‐RELATED PROTEIN KINASE 2 (SnRK2), has been demonstrated in cell culture assays under conditions of sucrose starvation (Kwiatkowski et al., 2024; Ramon et al., 2013; Van Dingenen et al., 2019). This interaction was observed in the cytosol of leaf mesophyll protoplasts (Van Dingenen et al., 2019). HXK1 also acts as a glucose sensor‐signalling molecule, directly linking the control of gene expression in the nucleus to the availability of glucose. Here, HXK1 has been shown to have nuclear functions complexing with VACUOLAR H^+^ ATPASE SUBUNIT B1 (VHA‐B1) and REGULATORY PARTICLE 5B (RPT5B), an AAA‐ATPase proteasome subunit, and with SWINGER (SWN) and CURLY LEAF (CLF), which are two catalytic subunits of the Polycomb Repressive Complex 2 (Cho et al., 2006; Liu et al., 2022). In the latter complex, interactions mediated through evolutionarily conserved SANT domains in SWN and CLF facilitate the association of HXK1 with target chromatin regions. Integration of HXK1 into the complex is required for H3K27me3 modification and the glucose‐mediated repression of gene expression. SWN and CLF can bind to catalytically inactive HXK1 variants G104D (Gly104 to Asp104) and S177A (Ser177 to Ala177), which still possess the glucose‐binding site, indicating that the HXK1 interaction with CLF or SWN does not depend on its glucose metabolic activity (Feng et al., 2015; Liu et al., 2022). Additionally, these studies showed that both seedling developmental arrest and the suppression of *CHLOROPHYLL A/B BINDING 2 (CAB2)* and *CARBONIC ANHYDRASE 1 (CAA)* expression by exogenous glucose application are maintained in catalytic mutant lines, but not in *gin2/hxk1*, *vha‐B1, rpt5b, swn‐1, clf‐50* mutants (Cho et al., 2006; Liu et al., 2022; Moore et al., 2003). This highlights that these responses are mediated by the glucose sensor‐signalling function of HXK1.

Interestingly, although HXK1‐mediated responses such as developmental arrest and the suppression of *CAB2/CAA* expression are robustly triggered by externally applied glucose, the regulation of seedling hypocotyl elongation by HXK1 occurs under conditions of low light and reduced nutrient availability (Cho et al., 2006; Moore et al., 2003). Indeed, loss‐of‐function HXK1 mutants, glucose‐insensitive 2 (*gin2‐1*) (*Landsberg erecta*) and *hxk1‐3* (*Columbia‐0*), exhibit notably reduced hypocotyl length, particularly in limited light conditions (Jang et al., 1997; Kelly et al., 2021). Additionally, the *vha‐B1* and *rpt5b* mutants phenocopy the *gin2‐1* short hypocotyl phenotype, while the catalytically inactive alleles *hxk1* ^*G104D*^ and *hxk1* ^*S177A*^ restore the WT hypocotyl response (Cho et al., 2006; Moore et al., 2003). These findings indicate that the regulation of hypocotyl growth by HXK1 can be mediated through its nuclear signalling function. It would, however, be interesting to determine whether the catalytic activity of HXK1 is necessary for driving early seedling growth through the metabolism of seed reserves. This role could be important for supporting growth in environments with limited light, where the transition to photoautotrophic growth might be postponed.

PHYTOCHROME INTERACTING FACTORS (PIFs) are a family of basic helix–loop–helix (bHLH) transcription factors, known to have key roles in regulating hypocotyl elongation in low light and darkness. PIFs (PIF1, 2–8) are regulated by the phytochrome family of photoreceptors, of which phyB has been shown to be critically important in regulating seedling de‐etiolation (Cheng et al., 2021). Excitation of the phyB chromophore with red (600–700 nm) light induces a conformational change from the inactive (Pr) to the active (Pfr) form, which preferentially binds to PIFs. This physical interaction results in rapid phosphorylation and subsequent degradation of PIFs 1, 3, 4 and 5, as well as sequestering PIFs from their target promoters (Leivar, Monte, Al‐Sady, et al., 2008; Park et al., 2012; Park et al., 2018). As a result, these PIFs are most active in darkness and low light, evident in the short hypocotyl phenotype of the quadruple *pifQ* mutant, which comprises *pif1‐1, pif3‐1, pif4‐2* and *pif5‐3* alleles (Leivar, Monte, Al‐Sady, et al., 2008). Thus, light‐activated phyB repression of PIFs is critical for the switch from skotomorphogenic to photomorphogenic growth.

Both phytochrome and PIFs have been implicated in carbon resource partitioning, where they have an important role in adjusting growth allocations to sink tissues in darkness and shaded conditions (de Wit et al., 2016; Krahmer et al., 2021; Yang et al., 2016). PIFs are involved in sucrose regulation of hypocotyl growth; the *pifQ* mutant is insensitive to sucrose supplementation, while PIF4 and PIF5 protein abundances are moderated by sucrose (Liu et al., 2011; Pereyra et al., 2022; Stewart et al., 2011). PIF4 has an important role in coupling carbon status to temperature‐dependent hypocotyl growth (Hwang, Kim, et al., 2019; Hwang, Susila, et al., 2019; Bian et al., 2022). Here, Trehalose‐6‐phosphate (Tre6P), a key indicator of sucrose status, stabilises PIF4 by inhibiting KIN10‐mediated PIF4 phosphorylation and 26S proteasome destruction (Hwang, Kim, et al., 2019; Hwang, Susila, et al., 2019). Additionally, a link has been made between guard cell‐located PIF4 and HXK1 and sucrose‐mediated hypocotyl elongation in long days. This work demonstrated that HXK1 overexpression in guard cells enhances *PIF4* expression and hypocotyl elongation in response to blue light wavelengths (Kelly et al., 2021). Thus, numerous connections exist between PIFs and carbon‐sensing pathways.

In this study, we evaluated whether HXK1 plays a role in seedling growth through the utilisation of seed reserves. Genetic, pharmacological and molecular analysis point to an important role for HXK1 enzymatic function in supporting hypocotyl growth in darkness and light‐limiting conditions. Our mRNA‐seq data reveal HXK1 exerts wide‐ranging control of metabolic genes, including a branched‐chain amino‐acid pathway, which provides an alternative source of carbon in starvation conditions. The mRNA‐seq analysis further reveals that HXK1 and PIFs signals converge at critical components of light signalling. Additionally, we show that elements of the HXK1 sensor‐signalling pathway do not operate under light‐ and nutrient‐limiting conditions.

## RESULTS

### HXK1 supports post‐germinative hypocotyl growth in a G6P‐dependent manner

**Figure 1:**
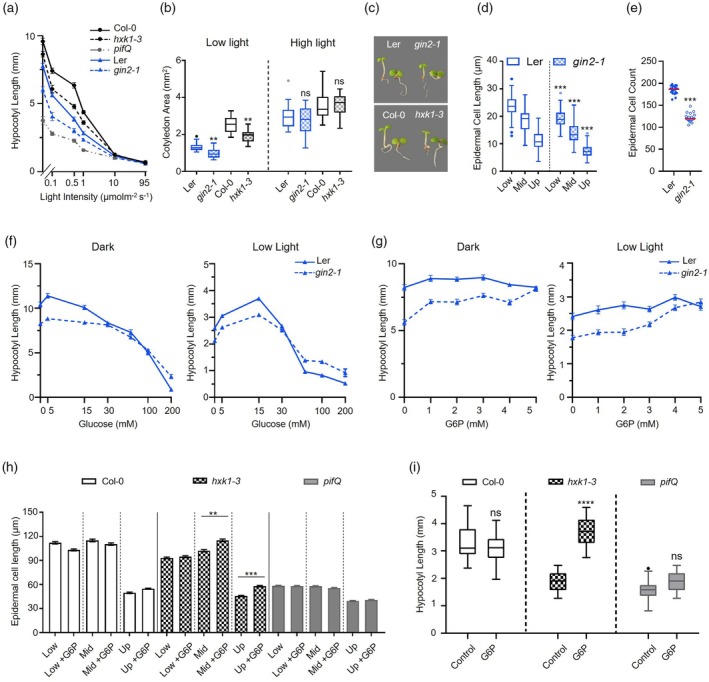
Analysis of the HXK1‐dependent hypocotyl growth phenotype and its dependence on G6P. (a) Hypocotyl fluence response curve for Ler WT, *gin2‐1*, Col‐0 WT, *hxk1‐3* and *pifQ* seedlings grown in darkness and continuous white light of increasing fluence rates. (b) Cotyledon area of seedlings grown in low (3 μmol m^−2^ s^−1^) or high (130 μmol m^−2^ s^−1^) fluence rates of continuous white light. (c) Images of representative seedlings from low (left) and high light (right). (d) Hypocotyl epidermal cell size in etiolated Ler WT and *gin2‐1* seedlings. (e) Cell counts in hypocotyl cell files in etiolated Ler WT and *gin2‐1* seedlings. (f) Glucose and (g) G6P dose–response curves for Ler WT and *gin2‐1* seedlings grown in darkness or low light (3 μmol m^−2^ s^−1^) SDs with increasing concentrations of glucose or G6P. (h) Epidermal cell length in lower (Low) middle (Mid) and upper (Up) regions of the hypocotyl in Col‐0 WT, *hxk1‐3* and *pifQ* seedlings grown in SDs with or without 5 mM G6P. (i) Hypocotyl length of seedlings grown in the same conditions as (h). In all the experiments seedlings were grown at 20°C. For a, f, g, h, error bars represent SEM. Data in boxplots (b, d, i) display the interquartile range (first‐third quartile), the median line, while whiskers extend 1.5 IQR beyond the quartiles. For b, d, e, h asterisks indicate significant differences according to the Student *t‐*test, as follows: *P < 0.05; **P < 0.01; ***P < 0.001; ****P < 0.0001, non‐significant (ns). For i, a Two‐way ANOVA test was used to obtain statistical significance (α = 0.05) between Control and G6P treatments, using Tukey's HSD post hoc test for multiple comparisons. Asterisks denote statistical significance, as follows: ****P < 0.0001, non‐significant (ns). All experiments comprise three biological replicates.

The *gin2‐1* mutant was previously reported to have impaired hypocotyl growth in low nutrient/low light conditions (Moore et al., 2003), which led us to examine the fluence rate range within which HXK1 operates. We established that the *gin2‐1* (Ler) and *hxk1‐3* (Col‐0) hypocotyls were shorter in darkness and very low irradiances of continuous light but were indistinguishable from the wild type (WT) at fluence rates greater than 10 μmol m^−2^ s^−1^ (Figure 1a). This altered fluence rate response is qualitatively similar to the quadruple *pifQ* (*pif1‐1, pif3‐1, pif4‐2, pif5‐3*) mutant, though less severe. To explore the light‐conditionality of the mutant phenotypes we grew *gin2‐1* and *hxk1‐3* in more natural photoperiodic conditions. We found both mutants displayed short hypocotyls in 8 h light (L), 16 h dark (D) short days (SDs), and also in longer photoperiods (16 h L, 8 h D), but only when the light fluence rate is low (Figure S1). Thus, the *gin2‐1* and *hxk1‐3* long hypocotyl phenotypes are evident in darkness and conditions when light availability is restricted. We also observe similar reductions in *gin2‐1* and *hxk1‐3* cotyledon area when light is limited (3 μmol m^−2^ s^−1^) but not in higher light conditions (100 μmol m^−2^ s^−1^) (Figure 1b,c).

To establish the cellular basis of the hypocotyl defect, we measured the epidermal cell length in the basal, middle and upper regions of the etiolated hypocotyl, and recorded the total cell number in epidermal cell files (Figure 1d,e). Our results show that *gin2‐1* has a lower cell file count and significantly shorter epidermal cells through the hypocotyl. Since hypocotyl cell number is largely determined during embryo formation, this suggests a role for HXK1 at this earlier developmental stage. However, given that some changes in cell numbers occur during post‐germination processes such as apical hook formation and stomata generation, it is conceivable that HXK1 might also facilitate cell division after germination (Saibo et al., 2003; Zadnikova et al., 2016). Nonetheless, as the primary mechanism behind hypocotyl elongation is the expansion of hypocotyl cells, our findings support a significant role for HXK1 in promoting the expansion of epidermal cells post‐germination, particularly in low‐light conditions (Gendreau et al., 1997).

Previously, HXK1‐dependent hypocotyl growth was shown to result from glucose‐induced nuclear signalling, rather than HXK1 enzymatic function (Cho et al., 2006; Moore et al., 2003). To explore this further, we examined the hypocotyl response over a range of glucose concentrations in low‐irradiance SDs and in darkness. In agreement with earlier findings, the WT glucose dose response under low light is biphasic; lower glucose concentrations stimulate hypocotyl growth, while higher concentrations are inhibitory (Singh et al., 2017; Figure 1f, Figures S2 and S3). In darkness, most doses inhibit hypocotyl growth, as the stimulatory phase peaks at low (0.5 mM) glucose concentrations. While the *gin2‐1* and *hxk1‐3* mutants respond to glucose in a similar manner to WT, their dose–response curves are slightly shifted, suggesting changed glucose sensitivity (Figure 1f, Figures S2c,d,g and S3). These response patterns are not evident in mannitol controls (Figure S2a,b,g,h). Notably, both *gin2‐1* and *hxk1‐3* exhibit significant reductions in the hypocotyl response at higher glucose concentrations, which aligns with the glucose‐insensitive phenotype reported for *gin2‐1* (Moore et al., 2003; Zhou et al., 1998). The altered mutant responses likely result from perturbed HXK1 signalling function, as previous studies have shown HXK1 nuclear signalling is triggered by similar glucose levels (Cho et al., 2006). For comparison, we administered increasing concentrations of glucose‐6‐phosphate (G6P), which is the product of HXK1 enzymatic activity. Contrasting with the glucose dose–response, the WT was largely insensitive to G6P application. Additionally, we observed that incremental rises in G6P dose progressively lengthened the hypocotyls of *gin2‐1* and *hxk1‐3*, which reached lengths comparable to the WT at 5 mM (Figure 1g, Figure S2e,f). These findings indicate that the phosphorylation of glucose to G6P by HXK1 is likely necessary to support hypocotyl growth in conditions of limited light and when external glucose is absent.

We next investigated the effects of G6P on epidermal cells located in lower, middle and upper regions of the hypocotyl in low‐light SDs. Similar to our results in dark conditions (Figure 1d,e), we found *hxk1‐3* had reduced cell length (Figure 1h). As before, WT seedlings were relatively insensitive to G6P, while this treatment enhanced epidermal cell length in the middle and upper regions and the overall length of the *hxk1‐3* hypocotyl (Figure 1h,i). As post‐germinative hypocotyl cell elongation proceeds in a basal to distal wave, this suggests a potential window of sensitivity to G6P (Boron & Vissenberg, 2014). Consistent with previous research, we found *pifQ* epidermal cells to be significantly shorter than those of WT (Martín et al., 2018). However, the *pifQ* mutant did not exhibit any changes in cell or hypocotyl length when treated with G6P, suggesting that the cell expansion defect in *pifQ* is not linked to G6P deficiencies (Figure 1h,i). Collectively, our data point to a role for HXK1 enzymatic activity in promoting hypocotyl elongation in darkness or conditions of low‐light availability.

### mRNA‐seq reveals a role for HXK1 in translation and carbon stress metabolism

**Figure 2:**
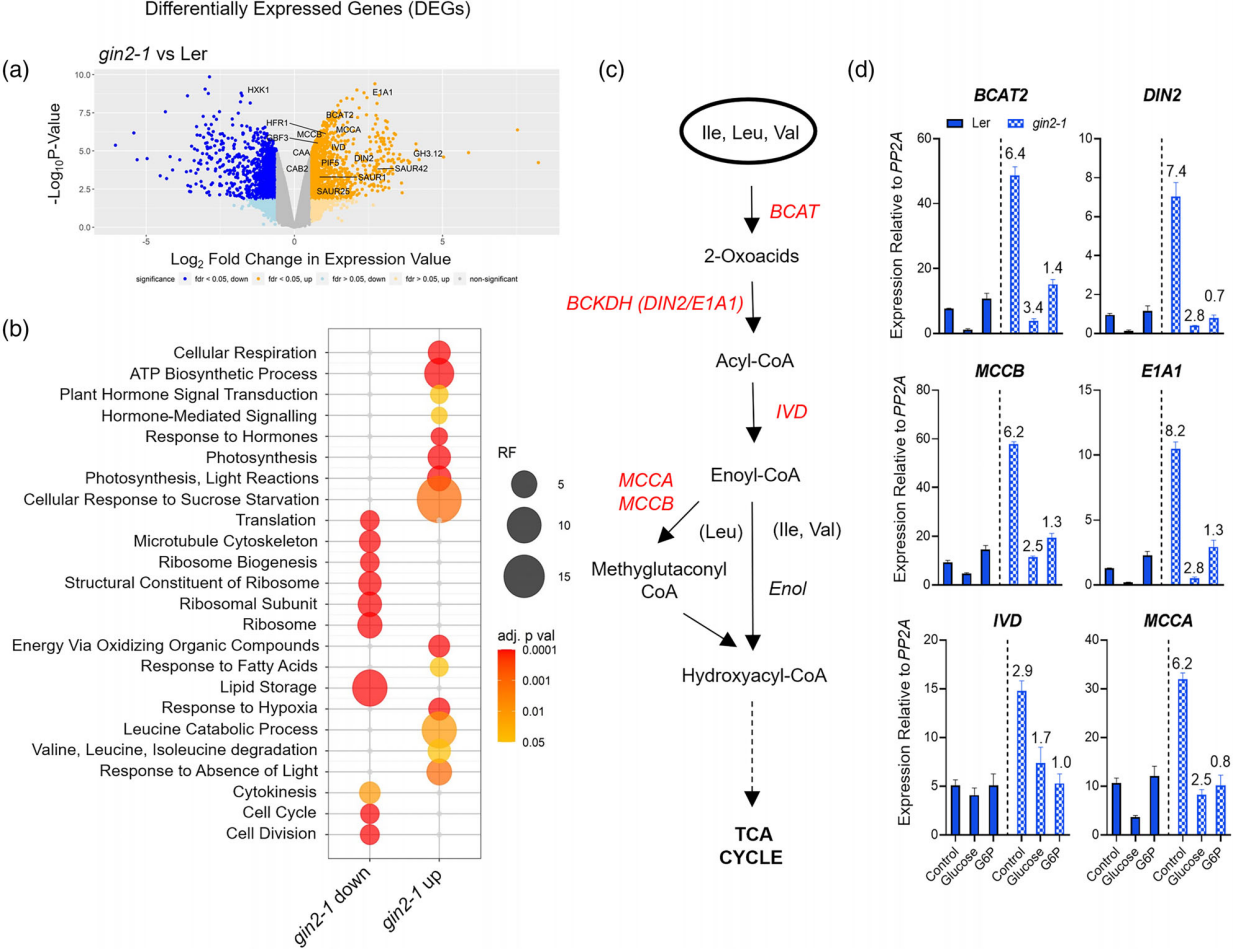
*gin2‐1* sequencing data reveals importance of HXK1 in translation and carbon stress metabolism in etiolated seedlings. (a) mRNAseq volcano plot. Yellow points indicate upregulation (log2FC > 0.58, corresponding to a fold change > 1.5), while blue points indicate downregulation (log2FC < −0.58). Statistical significance (FDR < 0.05) is represented by darker shading, whereas lighter shades denote non‐significant points (FDR ≥ 0.05). (b) Bubble plot of selected GO terms in mRNAseq. Genes of interest were collected and displayed as described in the materials and methods. The Hypergeometric Test with Benjamini–Hochberg correction was applied to determine over‐represented genes. Yellow to red colour scale represents *P* value (*P* val) scores (*P* < 0.05; *P* ≤ 0.01; *P* ≤ 0.001; *P* ≤ 0.0001; ns terms appear in grey colour), and the size of the bubble represents the representation factor (RF). (c) Diagram depicting branched chain amino acid (BCAA) catabolism in Arabidopsis (adapted from Neinast et al., 2019). *BCAA* enzyme genes identified by mRNAseq as *gin2‐1* regulated are shown in red. (d) *BCAA* gene expression is *gin2‐1* and G6P‐dependent. Transcript abundance of *BCAA* pathway genes (relative to *PP2A*), was determined by qPCR in etiolated WT and *gin2‐1* seedlings grown with or without 28 mM (0.5% w/v) glucose or 5 mM (0.125% w/v) G6P. Data are presented as mean values from biological triplicates (each with 3 technical replicates) ± SEM. Numbers shown represent fold‐change between *gin2‐1* and WT in equivalent treatments.

To gain a more in‐depth understanding of the HXK1 function in seedling establishment, we performed an mRNA‐seq on 4‐day‐old etiolated Ler and *gin2‐1* plants. Briefly, total RNA was extracted, and 6 stranded libraries were prepared from polyA purified RNA. These were pooled and sequenced on an Illumina HiSeq 4000 platform. Gene counts were extracted using the ASpli R package (Mancini et al., 2021) and the AtRTDv2 annotation (Zhang et al., 2017). Raw counts were filtered to remove lowly expressed genes, normalised to library size and gene expression was quantified using EdgeR (Robinson et al., 2010). This resulted in 21 256 genes (62% of the 34 212 annotated AtRTD2 genes) being considered for downstream analysis. Using custom R scripts, we performed differential gene expression (DGE) analysis using EdgeR, followed by gene ontology (GO) enrichment analysis. We discovered 2344 genes were significantly misregulated (|logFC| >0.58 and FDR <0.05) in *gin2‐1* compared to Ler WT, with a similar proportion of genes down (1156) and upregulated (1188) (Figure 2a; Table S1). When we evaluated these genes, we found that the downregulated category of genes included lipid storage and energy‐consuming processes, such as ribosome biogenesis and cell proliferation (Figure 2b). In this figure, the bubbles represent each GO term, the size of the bubble indicating the representation factor (RF) and the colour indicating the *P‐*value score. In contrast, the upregulated category had strong enrichment for genes involved in the cellular response to starvation, cellular respiration, ATP biosynthesis, amino acid catabolism and photosynthesis. These patterns are consistent with a starvation‐type response and a reduced capacity to catabolise sugars.

We noted that genes that were strongly upregulated in *gin2‐1* included several enzymes involved in the catabolism of the branched‐chain amino acid (*BCAA*) genes: *BRANCHED‐CHAIN AMINO ACID TRANSAMINASE 2* (*BCAT2*), *DARK INDUCIBLE 2* (*DIN2*), AT1G21400 (*E1A1*), *ISOVALERYL‐COA‐DEHYDROGENASE* (*IVD*), AT1G03090 (*MCCA*) and *3‐METHYLCROTONYL‐COA CARBOXYLASE* (*MCCB*) (Figure 2c). *BCAA* catabolism genes are often suppressed in carbon‐rich conditions and induced by stress or starvation, as they provide alternative substrates for respiration (Binder, 2010; Pires et al., 2016). Indeed, we observe strong glucose‐induced suppression of the majority of these genes in both the WT and *gin2‐1* (Figure 2d). The retention of the glucose response in *gin2‐1* could suggest that glucose‐activated HXK1‐nuclear signalling plays a minimal role in regulating these *BCAA* genes, although a compensatory role for HXK2 cannot be ruled out. Conversely, G6P application to WT has little to no impact on *BCAA* catabolism gene expression, yet it effectively restores *BCAA* expression to WT levels in *gin2‐1* mutants (Figure 2d). These G6P‐dependent trends mirror the effects observed in hypocotyl growth (Figure 1g; Figure S2e). Our findings therefore identify *BCAA* catabolism genes as targets of the HXK1‐G6P pathway that are suppressed upon its activation.

**Figure 3:**
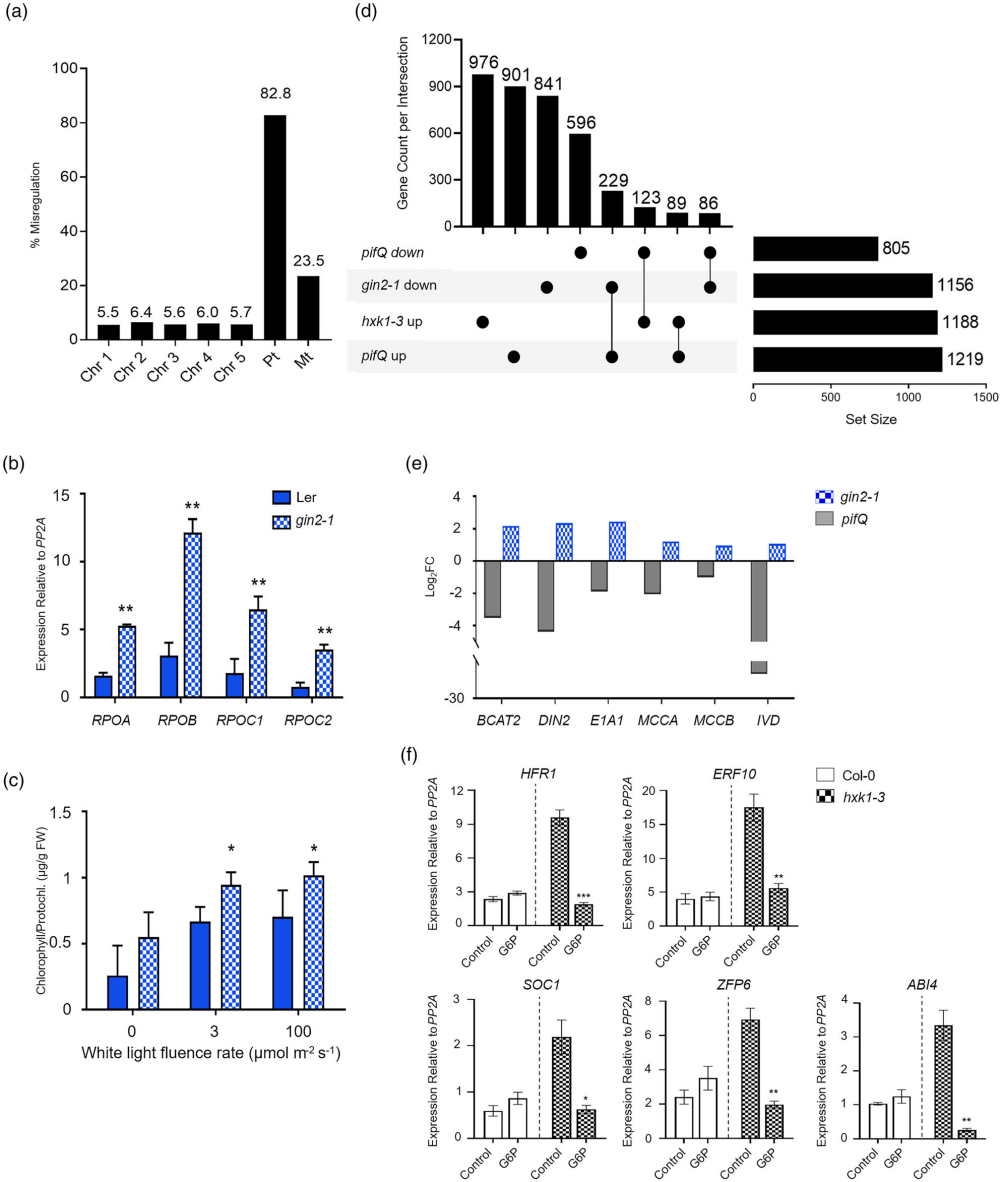
mRNA sequencing of *gin2‐1* highlights its critical role in the regulation of the plastid genome and demonstrates HXK1‐ and PIF‐ signalling intersect. (a) Percentage of chromosome, plastid (Pt) and mitochondrial (Mt) genes misregulated in *gin2‐1* compared to Ler WT. Numbers shown above bars represent the percentage of genes that are significantly (*P* ≤ 0.01) misregulated. (b) The impact of *gin2‐1* on the expression of chloroplast‐encoded *RNA POLYMERASE SUBUNIT* genes *RPOA, RPOB, RPOC1* and *RPOC2*, determined by qPCR. Seedlings were grown in darkness, and sampled on day 4. (c) Chlorophyll/protochlorophyllide content of *gin2‐1* and WT seedlings. Seedlings were grown at 20°C in darkness, then sampled after 1 h exposure to 3 or 100 μmol m^−2^ s^−1^ white light. Dark samples were harvested at the same time. (d) Transcriptome comparison between *gin2‐1* and *pifQ*. A comparison of misregulated genes in *gin2‐1* and *pifQ* (from Zhang et al., 2013) is presented, categorised into both common and exclusive subgroups. Numbers above the bars represent total counts. (e) Opposing *BCAA* gene expression in *gin2‐1* and *pifQ* (derived from mRNAseq data). (f) *HFR1, ERF10, SOC1, ZFP6* and *ABI4* expression, relative to *PP2A* (determined by qPCR), in dark‐grown Col‐0 (WT) and hxk1‐3 seedlings +/− 5 mM G6P, sampled on day 4. For b and c, data are presented as mean values ± SD, For f, data are presented as mean values ± SEM, and asterisks indicate significant differences (*gin2‐1* vs WT or Control versus G6P) according to Student *t‐*test, as follows: *P < 0.05; **P < 0.01; ***P < 0.001.

Another feature of the transcriptomic data is the extent to which HXK1 controls organellar gene expression, with 23.5% of mitochondrial and 82.8% of chloroplast genes upregulated in *gin2‐1* (Figure 3a). In the mitochondria, we observe enrichment for genes regulating electron transport and ATP synthesis. In the chloroplast, we see enrichment in all genes except for 23 genes associated with vesicle formation and chromatin remodelling. Figure 3b shows qPCR verification for the chloroplast‐encoded *RNA POLYMERASE SUBUNIT* genes *RPOA, RPOB, RPOC1* and *RPOC2*, that are required for chloroplast gene transcription. Given the dramatic effect on the chloroplast genome, we wanted to establish whether HXK1 affected seedling greening capacity. This we tested by growing seedlings in darkness for 4 days and then exposing them to 3 or 100 μmol m^−2^ s^−1^ white light for 1 h to promote chlorophyll production. We found *gin2‐1* has comparable levels of protochlorophyllide to WT in darkness, and slightly higher chlorophyll levels after exposure to light. Thus, light‐induced photosynthetic pigment production is enhanced in HXK1‐deficient seedlings, possibly as a compensatory response to constrained growth resources (Figure 3c).

### Transcriptomics data show HXK1 and PIF signalling converge at key light pathway genes

As HXK1 and PIFs both regulate hypocotyl elongation, albeit through different mechanisms, we wanted to establish if there was evidence for pathway crosstalk or convergence. By comparing transcriptomic data from our mRNA‐seq with an etiolated, 4‐day‐old *pifQ* mRNA‐seq dataset from Zhang et al. (2013), we discovered that there are 527 genes commonly misregulated between the two mutants: 175 of which are misregulated in the same direction, and 352 of which are misregulated in opposite ways (Figure 3d). Interestingly, for genes that are downregulated in *gin2‐1* and *pifQ (gin2‐1/pifQ down/down)* we observe enrichment (42 genes, or 24% of genes with synchronous regulation) in lipid storage, while in the *gin2‐1*/*pifQ* up/up category, there is significant enrichment (18 genes, or 10% of genes with synchronous regulation) in hypoxia‐response genes (Figure S4). Genes with opposing *gin2‐1/pifQ* down/up regulation are enriched in GO‐terms groups for translation, ribosome biogenesis, and cell proliferation. In contrast, opposing *gin2‐1/pifQ* up/down category genes include those involved in the cellular response to starvation and amino acid catabolism. Notably, this latter group includes the *BCAA* catabolism genes *BCAT2, DIN2, E1A1, IVD, MCCA*, and *MCCB* that we have identified as HXK1‐G6P regulated (Figure 2c), all of which show opposing regulation by HXK1 and PIFs (Figure 3e).

We also found *gin2‐1* up‐regulated genes were enriched for hormone categories, a proportion (20.5%) of which were also down regulated in *pifQ* (Figure 2b, Figure S4). Amongst the antagonistically regulated genes are *ETHYLENE RESPONSE FACTOR 10 (ERF10), NAC‐LIKE, ACTIVATED BY AP3/PI (NAP), TCP DOMAIN PROTEIN 12 (TCP12), LONG HYPOCOTYL IN FAR‐RED (HFR1) and the ABA‐responsive G‐BOX BINDING FACTOR 3 (GBF3)* (Table S2). Additionally, the *gin2‐1* upregulated (only) category includes a number of genes with links to phytochrome or PIF signalling, such as *HEAT SHOCK FACTOR A6A (HSFA6A), SUPPRESSOR OF OVEREXPRESSION OF CO 1/AGAMOUS‐LIKE 20 (SOC1/AGL20), REVEILLE 7 (RVE7), RVE4, ZINC FINGER PROTEIN 6 (ZFP6), ABA INSENSITIVE 4/GLUCOSE INSENSITIVE 6 (ABI4/GIN6), B‐BOX DOMAIN PROTEIN 17 (BBX17), BBX27* and *BEL1‐LIKE HOMEODOMAIN 1 (BLH1)* (Table S2) (Halliday et al., 2003; Staneloni et al., 2009; Gray et al., 2017; Ramegowda et al., 2017; Wu et al., 2018; Zhang et al., 2019; Barros‐Galvão et al., 2020; Tian et al., 2022; Cota‐Ruiz et al., 2022; Luo et al., 2024). Thus, HXK1 and PIF signalling appear to intercept at key nodes in the light and hormonal regulatory network.

To explore whether the influence of HXK1 on light pathway genes is linked to its catalytic function, we performed qPCR on *HFR1* and *ERF10*, from the antagonistically regulated category, alongside *SOC1, ZFP6* and *ABI4*, which were identified as upregulated in *gin2‐1* (Figure 3f). The qPCR results corroborate our *gin2‐1* mRNAseq data, as *hxk1‐3*, exhibits increased gene expression relative to WT. Additionally, they demonstrate that treating *hxk1‐3* with G6P reverses this elevated expression, whereas the WT remains unaffected by G6P treatment. These findings suggest that the enzymatic activity of HXK1 may play a role in the regulation of genes associated with light signalling and photomorphogenic growth.

### HXK1‐regulated hypocotyl growth on glucose‐free media does not require nuclear HXK1 partners

**Figure 4:**
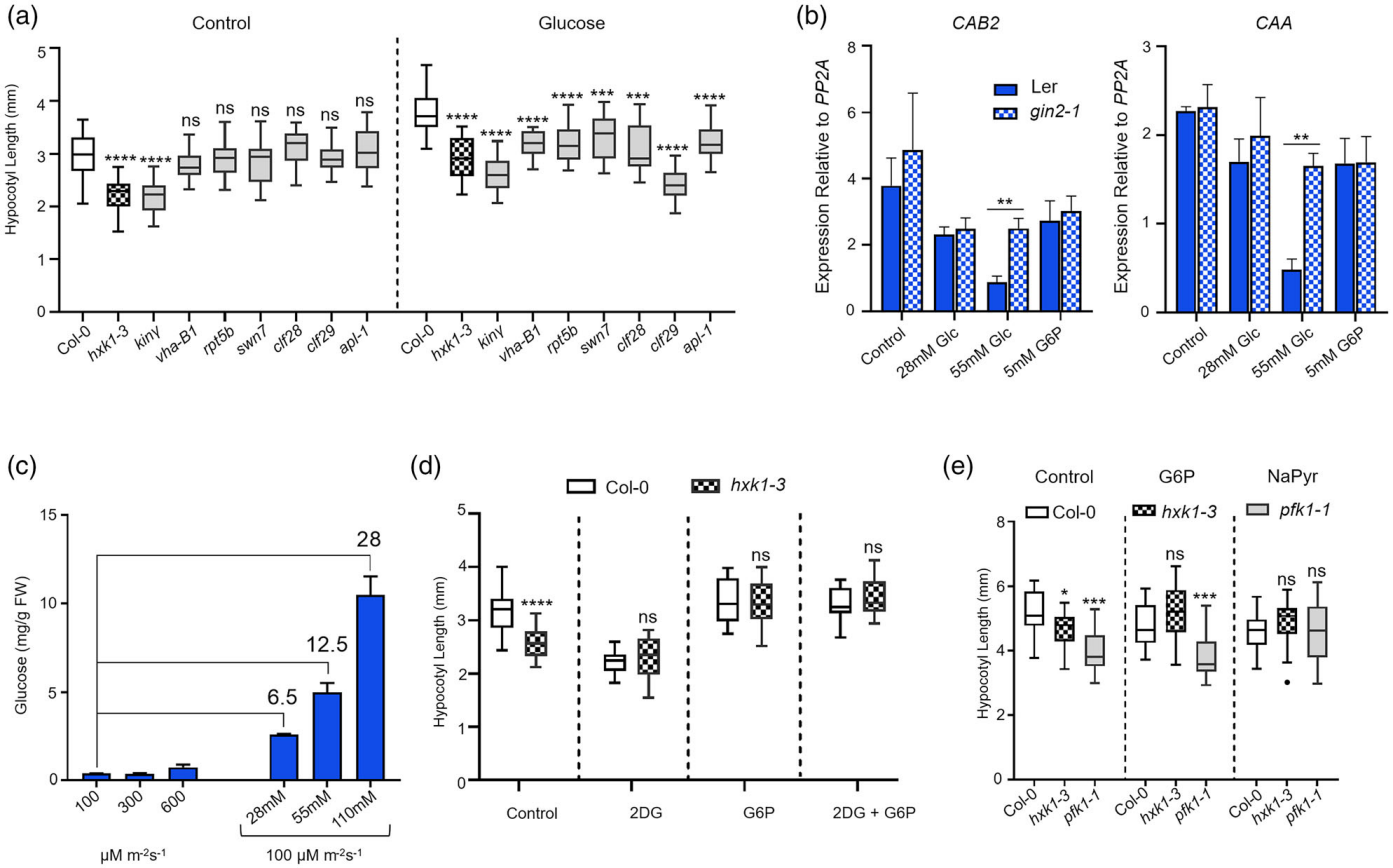
The short hypocotyl phenotypes of HXK1 nuclear signalling mutants require topical glucose application. (a) Hypocotyl length of Col‐0, *hxk1‐3*, *vha‐B1, rpt5b, swn‐7, apl‐1, clf28* and *clf29* grown in low light (3 μmol m^−2^ s^−1^) SDs without and with 28 mM glucose. (b) HXK1 regulation of *CAB2* and *CAA* expression is glucose‐dependent. *CAB2* and *CAA* expression (relative to *PP2A*) is shown for Ler and *gin2‐1*, in control conditions and when treated with 28 mM glucose, 55 mM glucose, or 5 mM G6P. (c) Internal glucose concentrations of Ler WT seedlings grown in white light of 100, 300, or 600 μmol m^−2^ s^−1^ or under 100 μmol m^−2^ s^−1^ light supplied with 28, 55 or 110 mM glucose. For b and c, seedlings were sampled at ZT4 on day 4. (d) Hypocotyl length of Col‐0 and *hxk1‐3* seedlings grown without or with 0.05 mM of 2‐deoxy‐D‐glucose (2DG), 5 mM G6P or 2DG + G6P. (e) (Na)Pyruvate complements the *hxk1‐3* phenotype. Hypocotyl length of Col‐0, *hxk1‐3* and glycolysis enzyme mutant *pfk1‐1*, in control conditions, with G6P or with sodium pyruvate (NaPyr). In all experiments seedlings were grown in 20°C SDs (3 μmol m^−2^ s^−1^ white light [except c]) for 4 days. Data in boxplots (a, d, e) display the interquartile range (first‐third quartile), the median line, while whiskers extend 1.5 IQR beyond the quartiles. A Two‐way ANOVA test was used to obtain statistical significance (α = 0.05), using Tukey's HSD post hoc test for multiple comparisons. Asterisks denote statistical significance as follows: *P < 0.05; **P < 0.01; ***P < 0.001; ****P < 0.0001, non‐significant (ns). For b and c, data are presented as mean values ± SD, and SEM, respectively. Asterisks indicate significant differences according to Student *t‐*test, as follows: **P < 0.01.

Our mRNA‐seq data indicate that in light‐restricted conditions, HXK1 has a role in controlling nutrient resource management, while our physiological data implicate HXK1‐G6P function in stimulating hypocotyl growth. Previous research demonstrated that mutant alleles of VHA‐B1 and RPT5B, which form a nuclear complex with HXK1, impair hypocotyl growth under low‐light conditions (15 μmol m^−2^ s^−1^) and moderate glucose concentrations (11.2 mM, 0.2% w/v) (Cho et al., 2006). Consistent with these findings, we observed that the *vha‐B1* and *rpt5b* mutants displayed a shortened hypocotyl phenotype under the same conditions as Cho et al. (2006) as well as under higher glucose concentrations (28 mM, 0.5% w/v), but not in the absence of glucose (Figure 4a; Figure S5a). This observation implies that, in contrast to *gin2‐1/hxk1‐3*, the short hypocotyl phenotype in *vha‐B1* and *rpt5b* is contingent on the presence of exogenous glucose. Qualitatively similar results were observed for *swn‐7, clf28, clf29*, and *apl‐1*, mutants of PRC2, which is known to complex with HXK1 (Liu et al., 2022). Contrasting with the mutations affecting nuclear‐located components, and analogous to *hxk1‐3*, the cytosolic *kinγ* mutant exhibited a short hypocotyl phenotype irrespective of glucose levels (Figure 4a) (Van Dingenen et al., 2019). These data suggests that under conditions of low light, the levels of internal glucose might be too low to trigger the HXK1 nuclear signalling pathway that regulates hypocotyl elongation. However, KINγ, which is located in the cytosol, is able to perform this regulatory function even in the absence of glucose.

### HXK1 represses *CAB2* and *CAA* expression in response to high glucose levels

Earlier reports implicated HXK1 (VHA‐B1 and RPT5B) in glucose repression of *CAB2* and *CAA* expression, so we were interested to establish if this regulation was exclusively through the HXK1 glucose signalling route (Cho et al., 2006). In line with these studies, we observe HXK1‐dependent repression of *CAB2/CAA* when seedlings are grown in the presence of 55 mM (1% w/v), but not 28 mM (0.5% w/v) glucose (Figure 4b). The 28 mM and 55 mM application doses lead to 6.5‐ and 12.5‐fold increases in internal glucose levels, while a ~115 mM (2% w/v) leads to a 28‐fold increase compared to controls (Figure 4c). We noted that the lowest concentration (28 mM) greatly exceeds glucose levels in seedlings exposed to the higher light fluence rates of 300 or 600 μmol m^−2^ s^−1^. We also show that *CAB2* and *CAA* expression is not substantially affected by *gin2‐1* following G6P application. Consequently, our data supports the notion that *CAB2* and *CAA* are suppressed by HXK1‐VHA‐B1‐RPT5B in response to glucose application (Cho et al., 2006; Moore et al., 2003). However, as with the response observed in hypocotyls (Figure 4a), the endogenous glucose levels present under light‐limited conditions fail to activate this glucose signalling response.

### Metabolisable G6P is required to support hypocotyl growth in low‐light conditions

To further investigate the HXK1 enzymatic role in hypocotyl extension, we applied the glucose analogue 2‐deoxy‐D‐Glc (2DG), which can be phosphorylated by HXK1 but is not further metabolised (Jang & Sheen, 1994; Yamada & Mine, 2024). Therefore, 2DG effectively blocks the glycolytic pathway without disrupting glucose signalling. We found that under glucose‐free conditions, applying 2DG reduces WT hypocotyl growth to a length comparable to that of the 2DG‐resistant *hxk1‐3*. Additionally, administering G6P to 2DG‐treated WT hypocotyls restores them to their normal length (Figure 4d). This suggests that in light limiting conditions, metabolisable G6P is required for HXK1‐mediated hypocotyl growth. Additionally, we found that glucose application stimulated hypocotyl growth in both WT and *hxk1‐3*, even in the presence of 2DG, indicating that this growth response is mediated through a mechanism independent of G6P. (Figure S5b). Our data infers that post‐germination HXK1‐G6P plays an important role in facilitating seedling hypocotyl growth. To consolidate our findings, we analysed the mutant for phosphofructokinase (PFK1), which controls a rate‐limiting step in glycolysis (Mustroph et al., 2007). We found that in low light the *pfk1‐1* mutant phenocopies the *hxk1‐3* short hypocotyl, and as expected *pfk1‐1* is unresponsive to G6P but is rescued by the application of the glycolytic product, pyruvate (Figure 4e). These results further reinforce the important role of the HXK1, and the glycolytic pathway in supporting hypocotyl growth in light limiting conditions.

## DISCUSSION

### HXK1‐mediated hypocotyl elongation is facilitated through G6P provision

The Arabidopsis seedling hypocotyl is an excellent model system for the study of carbon resource management and regulation (Chen et al., 2018; Ivakov et al., 2017; Oh et al., 2014; Singh et al., 2017). Further, since post‐germinative cell expansion relies on the mobilisation and consumption of seed reserves, it offers a chance to explore the seedling system before photosynthesis begins (Gommers & Monte, 2018; Penfield et al., 2004; Zhao et al., 2018). In this study, we have shown that HXK1 plays a pivotal role in promoting cell expansion through its enzymatic function. Through seedling establishment, HXK1 operates as a master regulator of the plastome and controls multiple aspects of the metabolic starvation response. HXK1 signalling intersects with key genes in the light and hormone pathways, providing molecular routes to link carbon availability with growth regulation.

HXK1 is recognised to have two distinct roles: as an enzyme that catalyses the conversion of glucose to G6P, and as a nuclear‐located sugar sensor‐signalling molecule (Cho et al., 2006; Moore et al., 2003). Its role in seedling hypocotyl development has previously been ascribed solely to its nuclear signalling capacity. However, our data show that when glucose is not supplied exogenously, HXK1 supports hypocotyl growth through G6P provision (Figure 1g–i, Figure S2, Figure 4d,e). This regulation becomes apparent when light is limited, whether through a shorter light period in diurnal conditions or with reduced light fluences in both continuous light and diurnal cycles (Figure 1a,b, Figure S1). In contrast to glucose, where dose–response curves are qualitatively similar in WT and *gin2‐1*/*hxk1‐3*, G6P selectively restores the *gin2‐1*/*hxk1‐3* short hypocotyl to wild‐type length (Figure 1f–h, Figure S2). This HXK1–G6P elicited response is mediated in part through epidermal cell expansion (Figure 1h). These findings emphasise the importance of HXK1 in facilitating growth, especially under conditions of darkness or low light, where the establishment of seedlings relies on the nutrients from seed reserves.

### HXK1 regulates the carbon stress transcriptome

To gain a broader understanding of HXK1 on the transcriptome, we conducted mRNA‐seq analysis. We found *gin2‐1* has a transcriptome signature that is reminiscent of sucrose starvation. Earlier studies indicated sucrose starvation leads to strong upregulation of genes involved in sugar metabolism, photosynthesis, lipid metabolism and cellular respiration, while downregulated gene categories include lipid storage, cell proliferation and ribosomal processes (Contento et al., 2004; Nicolai et al., 2006). Our study reports a qualitatively similar pattern in the transcriptome for *gin2‐1* (Figure 2b). Amongst the *gin2‐1* upregulated genes are those implicated in BCAA catabolism (Figure 2b–d), a highly conserved mitochondrial pathway that can be induced by intense carbon starvation (Binder, 2010; Heinemann & Hildebrandt, 2021; Hildebrandt et al., 2015). Activation of BCAA catabolism, which produces acetyl‐CoA for the TCA cycle and electrons for ATP production in the mitochondria, offers an alternative energy source that can be used when carbohydrate availability is low. As G6P supplementation specifically rescues the expression of these genes, our data indicate *BCAA* catabolic genes are regulated through HXK1–G6P induced signalling. Interestingly, the expression of *BCAA* catabolism genes is known to be directly regulated by SnRK1 kinase‐bZIP complexes (Pedrotti et al., 2018). This evolutionarily conserved protein kinase complex acts to maintain homeostasis under nutrient‐stress conditions. As G6P has been shown to inhibit SnRK1 activity it will be interesting to establish whether HXK1–G6P regulates *BCAAs*, and potentially other genes, via the SnRK1 kinase‐bZIP mechanism (Henninger et al., 2022; Nunes et al., 2013).

### In light limiting conditions HXK1 exerts strong control over plastid and mitochondrial genomes

Our mRNA‐seq data reveals a significant effect of HXK1 on the expression of non‐nuclear genes. In dark grown seedlings, 82.8% of the chloroplast and 23.5% of the mitochondrial genes were elevated in *gin2‐1* (Figure 3a–c, Table S3). In cotyledon cells of dark‐grown seedlings, plastids develop into etioplasts, characterised by the presence of protochlorophyllide, a precursor that is transformed into chlorophyllide, then chlorophyll following light exposure (Pogson & Albrecht, 2011). Glucose application is known to elevate chlorophyll content of light grown seedlings (Kushwah & Laxmi, 2014). Our data show that in etiolated seedlings, *gin2‐1* elevates plastid gene expression boosting chlorophyll levels during de‐etiolation (Figure 3c). Thus, HXK1 deficiency appears to prime the cellular machinery for photosynthesis rather than reliance on limited seed reserves to fuel growth.

Chloroplast biogenesis is tightly controlled by nuclear signals, primarily through SIGMA (SIG) factors. SIGs provide an anterograde signal that synchronises expression of genes in both the nucleus and the plastids, ensuring coordinated development and function (Hwang et al., 2022). In darkness, PIFs suppress *SIG* expression, preventing plastid gene expression, while light deactivates PIFs, reversing this process. Given the extensive upregulation of the plastid genome in *gin2‐1*, it is possible that HXK1 operates through such an anterograde pathway.

During germination, the mobilisation of storage reserves is accompanied by mitochondrial reactivation to provide energy for seedling growth (Paszkiewicz et al., 2017). For instance, TAGs which are oxidatively degraded into citrate are fed into the tricarboxylic acid (TCA) cycle within the mitochondrial matrix to generate ATP and reducing power (Graham, 2008). We found that HXK1 loss raises the expression of mitochondrial genes involved in oxidative phosphorylation, including proton transport and ATP synthesis. This upregulation is accompanied by increased expression of nuclear genes regulating BCAA catabolism, which may serve to boost aerobic respiration in otherwise carbon‐depleted conditions.

### HXK1 and PIF signalling convergence points

The *pifQ* mutant is known to have a short hypocotyl phenotype, particularly in light restricted conditions (Leivar, Monte, Oka, et al., 2008; Shin et al., 2009). We found that contrary to *hxk1‐3*, G6P did not reverse the short hypocotyl phenotype in *pifQ* to WT length, suggesting that G6P is not a limiting factor for growth in *pifQ*. Studies have shown PIF4 and PIF5 levels are elevated by sucrose, and additionally, PIF4 is regulated by T6P‐KIN10 and HXK1 (Hwang, Kim, et al., 2019; Hwang, Susila, et al., 2019; Kelly et al., 2021; Lilley et al., 2012; Pereyra et al., 2022; Stewart et al., 2011). This suggests that while these PIFs are regulated by sugar signalling, they do not substantially affect HXK1 metabolic function.

To establish if HXK1 and PIF signalling converged we compared our *gin2‐1* mRNA‐seq data with published *pifQ* mRNA‐seq data from dark‐grown seedlings (Zhang et al., 2013). This analysis revealed that 22% of *gin2‐1* regulated genes were also regulated by PIFQ, indicating a level of signal convergence (Figure 3d–f; Figure S4). We were interested to find that *BCAA* catabolism genes, which are repressed by HXK1, were found to be activated by PIFs (Figure 2a–d). This activation may be indicative of resource limitations in etiolated seedlings where PIFs actively drive growth.

A number of light and/or hormone signalling genes were identified as upregulated in *gin2‐1*, some of which were subject to opposing regulation by *pifQ*. We discovered that for *HFR1, ERF10, SOC1, ZFP6* and *ABI4*, their elevated expression in *hxk1‐3* was very effectively suppressed by G6P (Figure 3f). This suggests their regulation by HXK1 can result from its catalytic function. These genes have diverse roles in light signalling, including seedling photomorphogenesis (*HFR1, ZFP6*), phyA‐promotion of seed germination (*ABI4*), and shade‐induced flowering (*SOC1*) (Barros‐Galvão et al., 2020; Cota‐Ruiz et al., 2022; Halliday et al., 2003; Hornitschek et al., 2009; Liu et al., 2021; Zhang et al., 2019). Further, HFR1, an atypical bHLH transcription factor, occupies a central role in light signalling, operating by moderating PIF activity through heterodimerisation (Fairchild et al., 2000; Hornitschek et al., 2009; Shi et al., 2013). Thus, HXK1 has the potential to influence PIF activity through the repression of *HFR1* expression.

Previous research has shown that PIFs are required for sucrose‐induced hypocotyl elongation, and similar to this study, *pifQ* has reduced sensitivity to sucrose application (Liu et al., 2011; Stewart et al., 2011). Application of sucrose increases the levels of PIF4 and PIF5 proteins, while TREHALOSE 6‐PHOSPHATE (T6P) specifically affects the abundance of PIF4 protein (Hwang, Kim, et al., 2019; Hwang, Susila, et al., 2019; Pereyra et al., 2022; Stewart et al., 2011). T6P promotes thermal hypocotyl growth by elevating PIF4, through inhibition of KIN10, a catalytic subunit of SnRK1, which destabilises PIF4 (Hwang, Kim, et al., 2019; Hwang, Susila, et al., 2019). Further, PIF4 located in guard cells operates downstream of HXK1 to regulate hypocotyl elongation under blue light (Kelly et al., 2021). These studies indicate that PIFs are involved in sugar signalling, aligning with our findings that the *pifQ* mutant demonstrates a severely impaired response to glucose (Figure 1h,i). It is evident that PIFs play a crucial role in linking growth with the availability of carbon resources. Furthermore, HXK1 signalling has the capacity to affect the expression of specific light regulated genes.

### HXK1 promotes post‐germinative seedling establishment through metabolising seed reserves

Previous research has shown glucose‐activated HXK1 signalling occurs within nuclear complexes that include VHA‐B1 and RPT5B, or the Polycomb Repressive Complex 2 components SWN and CLF (Cho et al., 2006; Liu et al., 2022). Indeed, we observed hypocotyl growth defects in *vha‐B1, rpt5b, swn‐7, apl‐1, clf28* and *clf29* following exogenous glucose application. Conversely, both *gin2‐1/hxk1‐3* and *kinγ* display a short hypocotyl phenotype both with and without glucose. Thus, our data demonstrate that HXK1 can influence hypocotyl growth through pathways both associated with and independent of these nuclear complex components (Figure 4a). It also suggests that KINγ may be a key element of the non‐nuclear pathway.

These findings align with qPCR analyses of *CAB2* and *CAA*, both of which are genes repressed by glucose in an HXK1, VHA‐B1‐ and RPT5B‐dependent manner (Cho et al., 2006). Interestingly, we only observed HXK1‐mediated repression at 55 mM glucose (which elevates internal glucose levels by 12.5‐fold) and not the lower concentration of 28 mM glucose (6.5‐fold increase) (Figure 4b). This suggests HXK1‐dependency is only observed when glucose levels exceed normal physiological levels, which could be indicative of a stress response. The ABA signalling genes *ABI4/GIN6* and *ABI5* have been shown to have key roles in abiotic stress and the glucose signalling response (Arenas‐Huertero et al., 2000; Bossi et al., 2009; Liu et al., 2018; Xiong et al., 2023; Song et al., 2024). Additionally, similar to VHA‐B1 and RPT5B, ABI4 has been shown to regulate glucose‐mediated repression of *CAB2* expression (Dijkwel et al., 1997; Koussevitzky et al., 2007). It will therefore be interesting to establish whether the HXK1‐VHAB1‐RPT5B complex operates with ABI4 or other ABA signalling components to regulate this response.

A significant finding of our research is that G6P can effectively counter the inhibitory effects of both *hxk1‐3* and non‐metabolisable 2DG on hypocotyl growth. Further, addition of (Na)Pyruvate effectively restores the short hypocotyl of *hxk1‐3* and the glycolysis mutant *pfk1‐1* to WT length. These insights emphasise the importance of HXK1‐mediated glycolytic catabolism in fuelling hypocotyl expansion under light‐limiting conditions. In early seedling development, sucrose produced from TAGs and starch feeds into glycolysis, which in turn allows for the production of energy, carbon skeletons and metabolites that are required for cell expansion and growth (Graham, 2008; Penfield et al., 2004). Indeed, *phosphoenolpyruvate carboxykinase 1 (pck1)*, which is deficient in gluconeogenesis, and *wrinkled 1 (wri1)*, characterised by decreased seed TAG and glycolysis, both exhibit impaired hypocotyl growth (Kucsynski et al., 2022; Penfield et al., 2004). Our data suggest HXK1 performs an important role in metabolising glucose derived from sucrose, which is produced from seed reserves.

In conclusion, this study has shown HXK1 performs an important role in post‐germinative hypocotyl growth by enabling the consumption of seed reserves prior to establishment of photosynthetic competence. By controlling the expression of enzymes in the BCAA catabolic pathway, HXK1 provides a route to generate alternative respiratory substrates when carbon resources are limited. HXK1 control of the plastome provides a means to link photosynthetic machinery establishment to carbon needs. While HXK1 control of light signalling components provides a potential route to couple resource availability to the regulation of growth.

## EXPERIMENTAL PROCEDURES

### Plant material, growth conditions and treatments

The wild‐type Arabidopsis thaliana accessions used in this study are Landsberg erecta (Ler) and Columbia‐0 (Col). Seeds for mutants *gin2‐1* (Ler), *hxk1‐3* (Col), *pfk1‐1* (Col), *vha‐B1* (Col, SALK_028728), *rpt5b* (Col, SALK_069366) and *kinγ* (Col, At3g48530) were obtained from The Nottingham Arabidopsis Stock Centre (NASC), UK. The *swn‐7*, *apl‐1*, *clf28* and *clf29* alleles were kindly donated by Professor Justin Goodrich (University of Edinburgh). For all experiments, seeds were surface sterilised with bleach and Triton X‐100 sown on 0.5X MS plates (0.8% agar, pH 5.7) and stratified in darkness for 2–3 days at 4°C. All plants were grown at 18°C for 4 days after stratification unless otherwise specified.

### Seedling hypocotyl length and cotyledon area measurements

Images of seedlings laid flat on growth media were used to quantify hypocotyl length and cotyledon area using ImageJ (NIH, Maryland, USA) and Adobe Photoshop CS6 (Adobe, California, USA), respectively. For glucose‐6‐phosphate (G6P) (Sigma G7879), sodium pyruvate (Sigma P2256) and 2‐deoxy‐D‐glucose (D8375 Sigma‐Aldrich) treatments, the required concentrations from filter sterilised stocks were added to sterilised media and seeds directly sown. Unless otherwise stated, all experiments were performed in triplicate with 40 seedlings per replicate.

### Seedling hypocotyl cell counts and length measurements

For determination of etiolated hypocotyl epidermal cell lengths/numbers, seedlings were mounted on slides with water and visualised using an Eclipse E600 Nikon DIC microscope at 20x magnification. Individual cell lengths from each section of the hypocotyl (basal, middle, upper) were measured using ImageJ, and cell number per file was obtained by manually counting cells from the root emergence point to the apical meristem. Unless otherwise stated, all experiments were performed in triplicate with at least 20 seedlings per replicate.

### Glucose and chlorophyll/protochlorophyllide quantification

Seedlings were harvested in liquid nitrogen, finely ground into a powder and ethanol extracted three times. Glucose was then quantified from ethanol extracts using enzymatic degradation at 340 nm wavelength and normalised to material fresh weight. Protochlorophyllide and chlorophyll were quantified from these extracts by absorption in the 640–670 range optimised for protochlorophyllide or chlorophyll detection. Unless otherwise stated, all experiments were performed in triplicate with 50 seedlings per replicate.

### Gene expression analysis

For qRT‐PCR experiments, seedlings harvested in liquid nitrogen were ground into fine powder. Total RNA was extracted using the RNeasy Plant Mini Kit (Qiagen) with on‐column DNase digestion. cDNA synthesis was performed using the qScript cDNA SuperMix (Quanta Biosciences) as described by the manufacturer. The qRT‐PCR was set up as a 10 μL reaction using SYBR^®^ Green 1480 Lightcycler^®^ Master (Roche) in a 384‐well plate, performed with a Lightcycler 480 system (Roche). Results were analysed using the Light Cycler 480 software. The primers used in this study are listed in Table S4. Unless otherwise stated, all experiments were performed in triplicate with 50 seedlings per replicate.

### cDNA library preparation and high throughput sequencing

Total RNA was extracted from 4‐day‐old etiolated Ler and *gin2‐1* seedlings (biological duplicates of 50 seedlings per replicate) as described above. Samples were then sent to Edinburgh Genomics (University of Edinburgh, UK) for QC check and sequencing. Briefly, quality check of the samples was performed using Qubit with the broad range RNA kit (Thermo Fisher Scientific) and Tapestation 4200 with the RNA Screentape for eukaryotic RNA analysis (Agilent). Libraries were prepared using the TruSeq Stranded mRNA kit (Illumina) and then validated. Samples were pooled to create 4 multiplexed DNA libraries, which were paired‐end sequenced on an Illumina HiSeq 4000 platform (Machine name K00166, Run number 346, flowcell AHT2HKBBXX, lanes 5 and 6). On average, 26.6 million 150 nt PE reads were obtained for each sample.

### Processing of mRNA sequencing reads

Raw sequence reads were trimmed with cutadapt 2.8 (Martin, 2011) with default parameters and ‐‐a set to ‘AGATCGGAAGAGC’, to eliminate adapter contamination from the PE reads. Trimmed reads were aligned against the Arabidopsis thaliana genome (TAIR10) with HiSat2 v2.2.1 (Kim et al., 2019) with default parameters, except in the case of the maximum intron length parameter, which was set at 5000. Count tables for the different feature levels were obtained from bam files using the ‘ASpli::readCounts()’ function of ASpli package version 2.4.0 (Mancini et al., 2021) with custom R scripts and considering the AtRTD2 transcriptome (Zhang et al., 2017) (Figure S7). Count tables at the gene level presented a good correlation overall between replicates and samples (Table S4). Raw sequences (fastq files) used in this paper have been deposited in the ArrayExpress (Kolesnikov et al., 2015) database at EMBL‐EBI (www.ebi.ac.uk/arrayexpress) under accession number E‐MTAB‐7654.

### Differential gene expression (DGE) analysis

DGE analysis was conducted using custom R scripts for 21 256 genes whose expression was above a minimum threshold level (10 counts in 0.7 of samples in the smallest group that express the gene). DGE was estimated using the edgeR package version 3.36.0 (Lun et al., 2016; Robinson et al., 2010), and resulting P values were adjusted using a false discovery rate (FDR) criterion. Genes with *P* < 0.05, FDR < 0.05 and an absolute log2 fold change > 0.58 were considered differentially expressed. Volcano plots, calculation and plot of chromosomal distributions and UpSet plots of differentially expressed genes (DEGs) were generated using R (Figures S6, S7). All R scripts were run using R version 4.1.1 and Bioconductor version 3.14.

### GO and KEGG metabolic pathway analysis

Gene set enrichment and KEGG pathway enrichment analysis were performed using a combination of custom‐written R scripts and the clusterProfiler package (Yu et al., 2012) version 4.2.2 of Bioconductor. The 21 256 expressed genes were used as the universe gene set. All KEGG pathways of *A. thaliana* were derived from the KEGG Pathway Database (https://www.kegg.jp; Kanehisa et al., 2012). Only the terms with *P* < 0.05 and FDR < 0.1 were further considered. Bubble plots were generated using R. All R scripts were run using R version 4.1.1 and Bioconductor version 3.14.

### Statistical analysis

The statistical difference between two populations was tested by two‐tailed, unpaired Student's *t*‐test. To compare three or more populations, a one‐way analysis of variance (ANOVA) followed by Dunnett's test (comparison against a control) was performed. All analyses were done using GraphPad Prism 7 (GraphPad Software) unless otherwise indicated.

## Accession numbers

Raw sequences (fastq files) used in this paper have been deposited in the ArrayExpress (Kolesnikov et al., 2015) database at EMBL‐EBI (www.ebi.ac.uk/arrayexpress) under accession number E‐MTAB‐7654. All custom R scripts are available at https://github.com/aromanowski/gin2_darkness. Alternatively, they are available upon request to the authors.

## Author Contributions

MZ, AG, ACL, AR and KJH planned and designed the research. AG designed and performed and analysed the mRNA‐seq experiment, AR conducted the bioinformatics analysis of the mRNAseq data. All other experiments were performed and analysed by MZ, AG, ACL and LDM. The authors MZ, AG, ACL, AR and KJH contributed to manuscript preparation.

## CONFLICT OF INTEREST

The authors declare no conflicts of interest.

## Supporting information


**Figure S1.** Hypocotyl length of *gin2‐1* and *hxk1‐3* in photoperiodic conditions. (a) Ler WT, *gin2‐1*, Col‐0 WT and *hxk1‐3* seedlings were grown in short day (SD), 8hL:16hD, 12hL:12hD and long day (LD) 16hL:8hD, with a white light fluence rate of 100 μmol m^−2^ s^−1^ at 20°C. (b) The effect of reducing the fluence rate of seedlings grown in LDs. Boxplots display the interquartile range (first‐third quartile), the median line, while whiskers extend 1.5 IQR beyond the quartiles.
**Figure S2.** Dose–response curves for glucose, G6P and mannitol osmotic controls. (a) Hypocotyl length of Ler and *gin2‐1* seedlings grown on increasing concentrations of mannitol. (c–h) Hypocotyl length of Col‐0 and *hxk1‐3* seedlings grown on increasing concentrations of glucose, G6P or mannitol. Seedlings were grown for 4 days in darkness or short‐day (SD) low light (3 μmol m^−2^ s^−1^), at 20°C. Data are presented as mean values ± SEM.
**Figure S3.** Effect of low doses of glucose on hypocotyl growth in Col‐0 *hxk1‐3* and WT seedlings. Seedlings were grown in short‐day(3 μmol m^−2^ s^−1^) white light (20°C) for 4 days with increasing levels of glucose. Data are presented as mean values ± SEM.
**Figure S4.** Bubble plot of selected GO terms provides a visual comparison of common and distinct *gin2‐1* and *pifQ* gene categories (from mRNAseq data). Genes of interest were collected and displayed as described in Figure 2 and the materials and methods section.
**Figure S5.** (a) Hypocotyl length of Col‐0, *hxk1‐3, vha‐B1* and *rpt5b* mutant seedlings grown for 4 days at 20°C in constant white light (15 μmol m^−2^ s^−1^) on lighter growth medium supplemented with 0.2% w/v glucose (as per Cho et al., 2006). (b) Seedlings grown for 4 days at 20°C in 8:16 SDs (white light, 100 μmol m^−2^ s^−1^) supplemented with 0.05 mM 2‐deoxy‐D‐glucose (2DG), 28 mM glucose (Glc), both (2DG + Glc), or neither (Control). Boxplots display the median line, while whiskers indicate Tucky method. For a, asterisks indicate significant differences according to Student *t‐*test, as follows: *P < 0.05; **P < 0.01; ***P < 0.001, ****P < 0.0001, ns, non‐significant. For (b), a Two‐way ANOVA test was used to obtain statistical significance (α = 0.05), using Tukey's HSD post hoc test for multiple comparisons: asterisks denote statistical significance as follows: *P < 0.05; **P < 0.01; ***P < 0.001, ****P < 0.0001, ns, non‐significant.
**Figure S6.** Data analysis pipeline of mRNAseq.
**Figure S7.** Pearson sample‐similarity correlation of *gin2‐1* and Ler samples used in mRNAseq.
**Table S1.** The proportions of mRNAseq *gin2‐1* up/down genes (compared to Ler WT), represented in Figure 3b.
**Table S2.** (a) Selected light regulated genes from ‘*gin2‐1* up, *pifQ* down’ (a), and ‘*gin2‐1* up only’ (b) mRNAseq data.
**Table S3.** Count tables at the gene level organised under chromosome and plastome. DEG, Differentially Expressed Genes.
**Table S4.** Primers used in the course of this study.

## Data Availability

The data that supports the findings of this study are available in the supplementary material of this article.
