# Supplementary material for: Hexokinase 1 facilitates post‐germinative seedling growth through its catalytic function
Source: Plant J. 2026 Jul 8;127(1):e70967. doi: 10.1111/tpj.70967 (PMC13345567; doi:10.1111/tpj.70967)
Supplement: Supplementary file 1 — Figure S1. Hypocotyl length of gin2‐1 and hxk1‐3 in photoperiodic conditions. (a) Ler WT, gin2‐1, Col‐0 WT and hxk1‐3 seedlings were grown in short day (SD), 8hL:16hD, 12hL:12hD and long day (LD) 16hL:8hD, with a white light fluence rate of 100 μmol m−2 s−1 at 20°C. (b) The effect of reducing the fluence rate of seedlings grown in LDs. Boxplots display the interquartile range (first‐third quartile), the median line, while whiskers extend 1.5 IQR beyond the quartiles. Figure S2. Dose–response curves for glucose, G6P and mannitol osmotic controls. (a) Hypocotyl length of Ler and gin2‐1 seedlings grown on increasing concentrations of mannitol. (c–h) Hypocotyl length of Col‐0 and hxk1‐3 seedlings grown on increasing concentrations of glucose, G6P or mannitol. Seedlings were grown for 4 days in darkness or short‐day (SD) low light (3 μmol m−2 s−1), at 20°C. Data are presented as mean values ± SEM. Figure S3. Effect of low doses of glucose on hypocotyl growth in Col‐0 hxk1‐3 and WT seedlings. Seedlings were grown in short‐day(3 μmol m−2 s−1) white light (20°C) for 4 days with increasing levels of glucose. Data are presented as mean values ± SEM. Figure S4. Bubble plot of selected GO terms provides a visual comparison of common and distinct gin2‐1 and pifQ gene categories (from mRNAseq data). Genes of interest were collected and displayed as described in Figure 2 and the materials and methods section. Figure S5. (a) Hypocotyl length of Col‐0, hxk1‐3, vha‐B1 and rpt5b mutant seedlings grown for 4 days at 20°C in constant white light (15 μmol m−2 s−1) on lighter growth medium supplemented with 0.2% w/v glucose (as per Cho et al., 2006). (b) Seedlings grown for 4 days at 20°C in 8:16 SDs (white light, 100 μmol m−2 s−1) supplemented with 0.05 mM 2‐deoxy‐D‐glucose (2DG), 28 mM glucose (Glc), both (2DG + Glc), or neither (Control). Boxplots display the median line, while whiskers indicate Tucky method. For a, asterisks indicate significant differences according to Student t‐test [file TPJ-127-0-s001.docx]

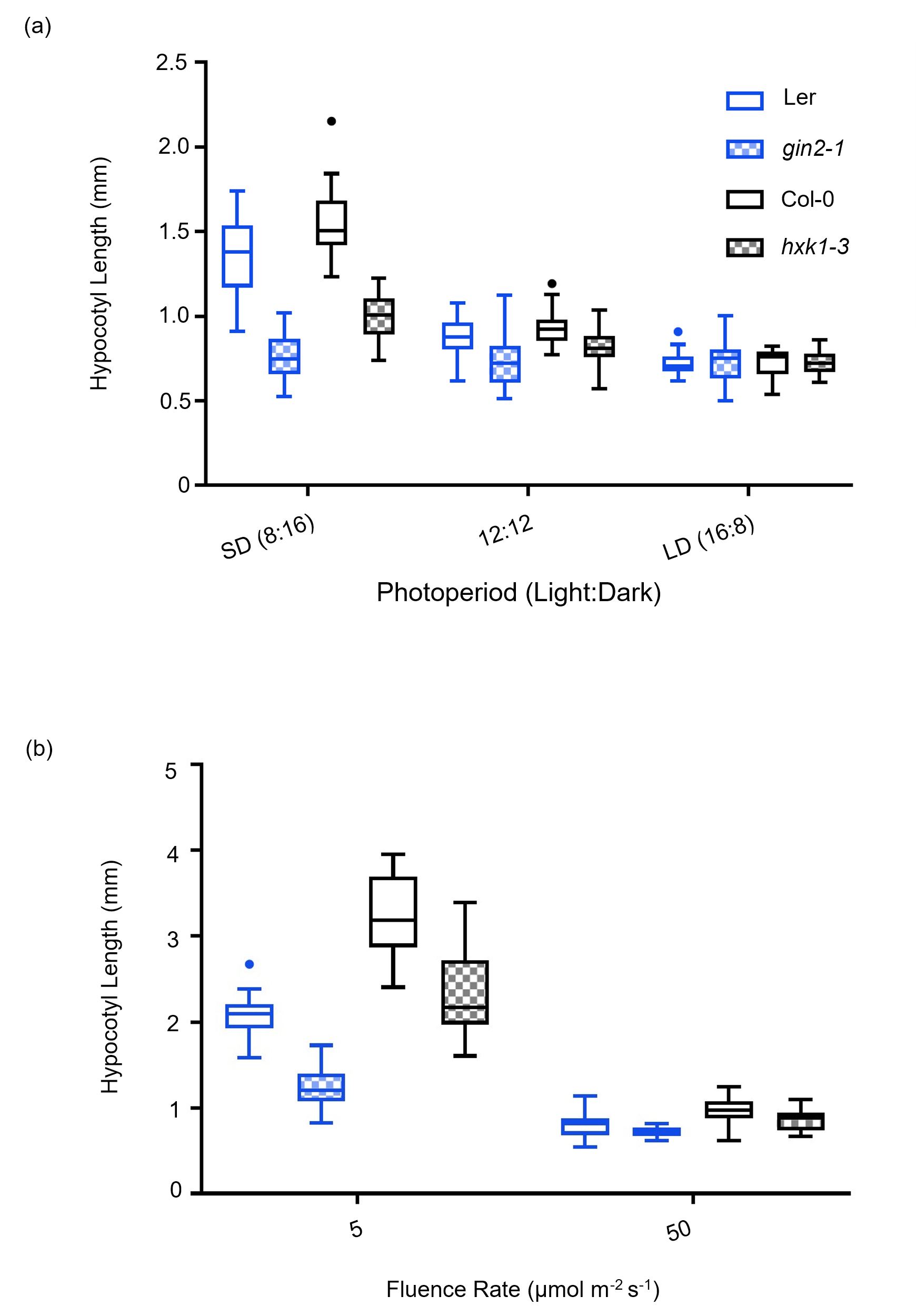


**Fig S1.** Hypocotyl length of *gin2-1* and *hxk1-3* in photoperiodic conditions. (a) Ler WT, *gin2-1*, Col-0 WT and *hxk1-3* seedlings were grown in short day (SD), 8hL:16hD, 12hL:12hD and long day (LD) 16hL:8hD, with a white light fluence rate of 100 µmol m^-2^ s^-1^ at 20℃. (b) The effect of reducing the fluence rate of seedlings grown in LDs. Boxplots display the interquartile range (first-third quartile), the median line, while whiskers extend 1.5 IQR beyond the quartiles.


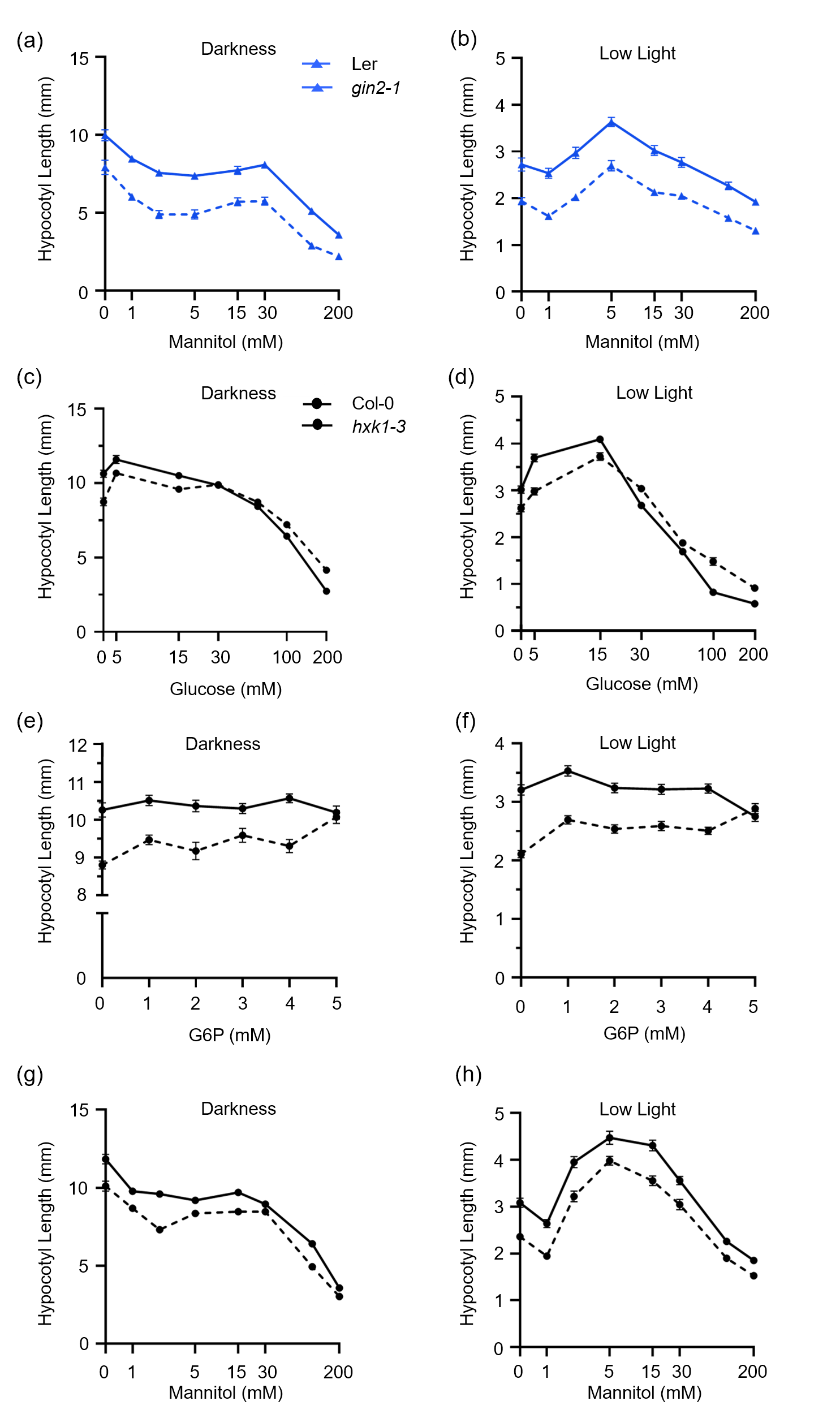


**Fig. S2** Dose response curves for glucose, G6P, and mannitol osmotic controls*.* (a) Hypocotyl length of Ler and *gin2-1* seedlings grown on increasing concentrations of mannitol. (c-h) Hypocotyl length of Col-0 and *hxk1-3* seedlings grown on increasing concentrations of glucose, G6P or mannitol. Seedlings were grown for 4 days in darkness or short-day (SD) low light (3 µmol m^-2^ s^-1^), at 20℃. Data are presented as mean values ± s.e.m.


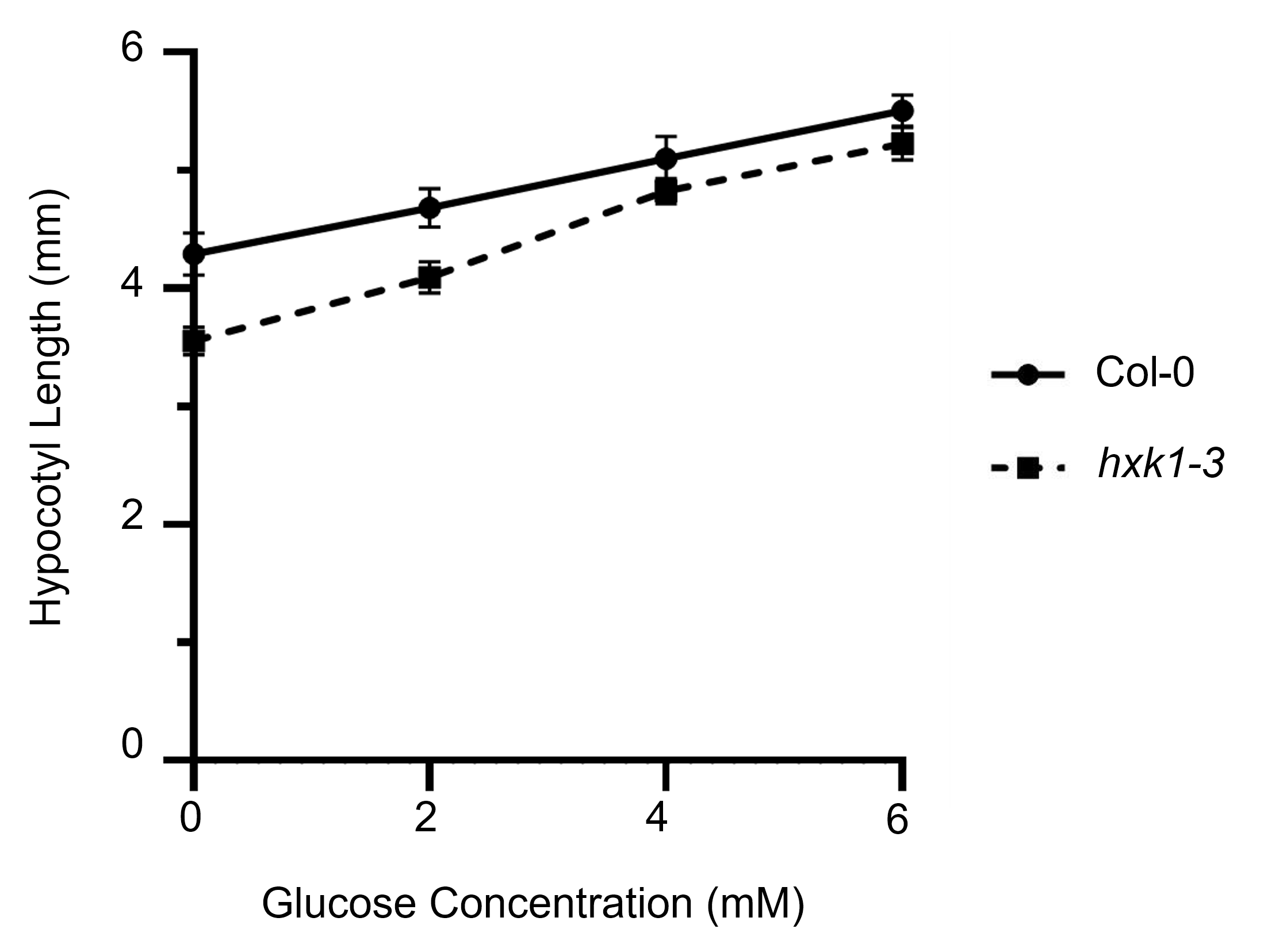


**Fig S3** Effect of low doses of glucose on hypocotyl growth in Col-0 *hxk1-3* and WT seedlings.Seedlings were grown in short-day(3 µmol m^-2^ s^-1^) white light (20℃) for 4 days with increasing levels of glucose. Data are presented as mean values ± s.e.m.


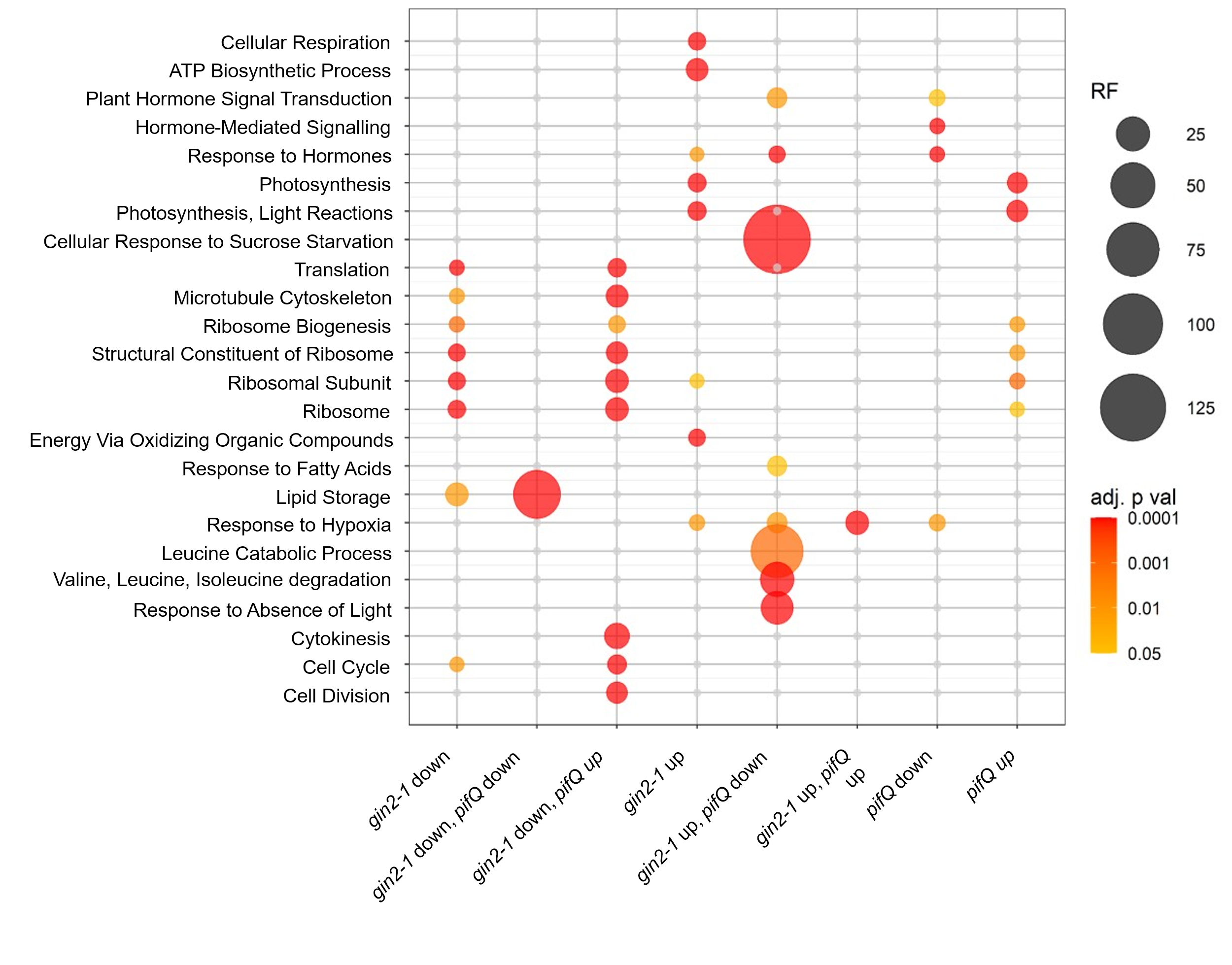


**Fig. S4** Bubble plot of selected GO terms provides a visual comparison of common and distinct *gin2-1* and *pifQ* gene categories (from mRNAseq data). Genes of interest were collected and displayed as described in Fig. 2 and the materials and methods section.


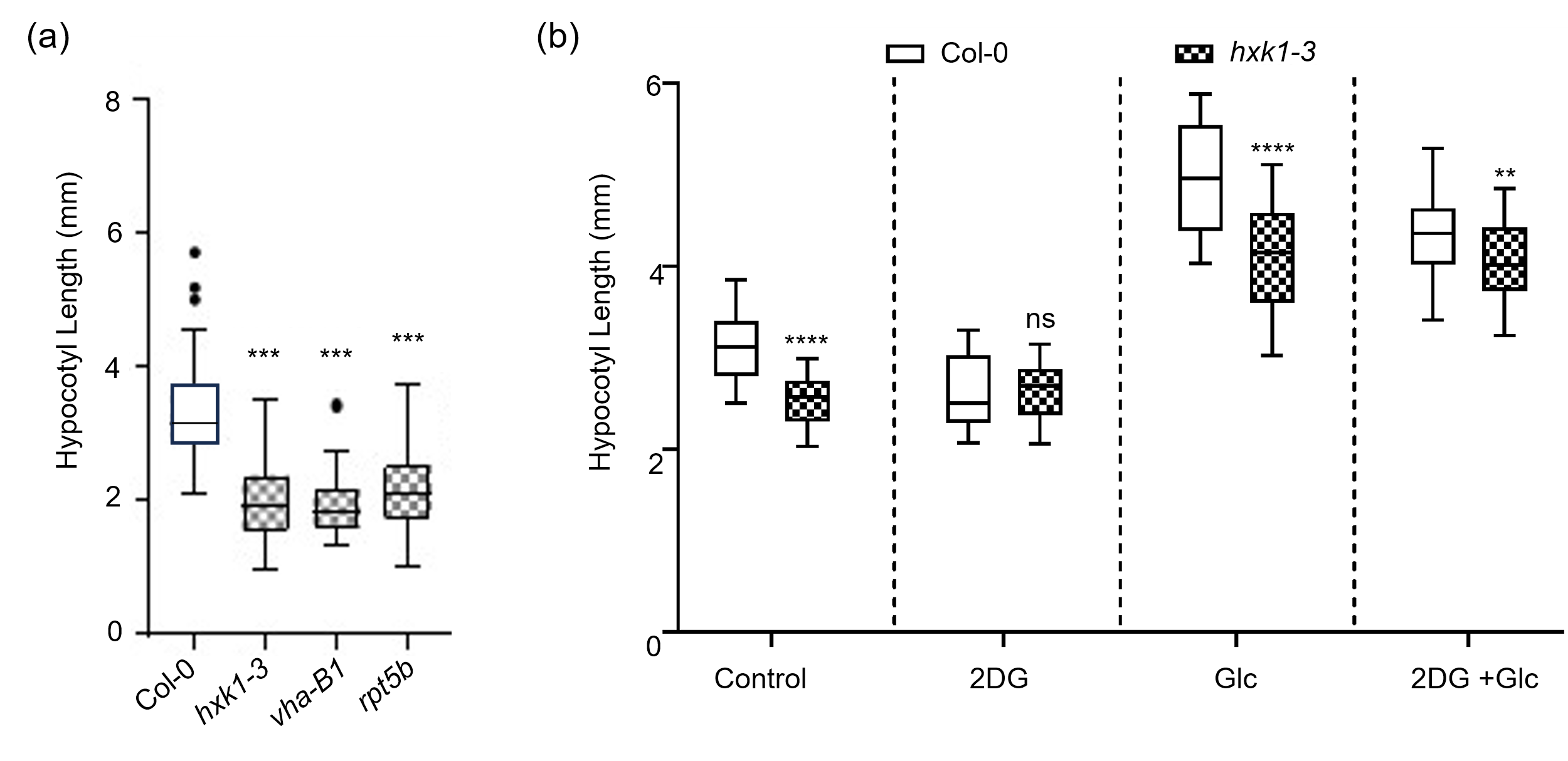


**Fig. S5** (a) Hypocotyl length of Col-0, *hxk1-3, vha-B1,* and *rpt5b* mutant seedlings grown for 4 days at 20°C in constant white light (15 µmol m^-2^ s^-1^) on lighter growth medium supplemented with 0.2% w/v glucose (as per Cho et al., 2006). (b) Seedlings grown for 4 days at 20 ℃ in 8:16 SDs (white light, 100 µmol m^-2^ s^-1^) supplemented with 0.05mM 2-deoxy-D-glucose (2DG), 28mM glucose (Glc), both (2DG+Glc), or neither (Control).

Boxplots display the median line, while whiskers indicate Tucky method. For a, asterisks indicate significant differences according to Student *t-*test, as follows: **P*<0.05; ***P*<0.01; ****P*<0.001, *****P*<0.0001, ns, non-significant. For b, a Two-way ANOVA test was used to obtain statistical significance (α=0.05), using Tukey’s HSD posthoc test for multiple comparisons**:** asterisks denote statistical significance as follows: *** *P*<0.05; ***P*<0.01; ****P*<0.001, *****P*<0.0001, ns, non-significant.


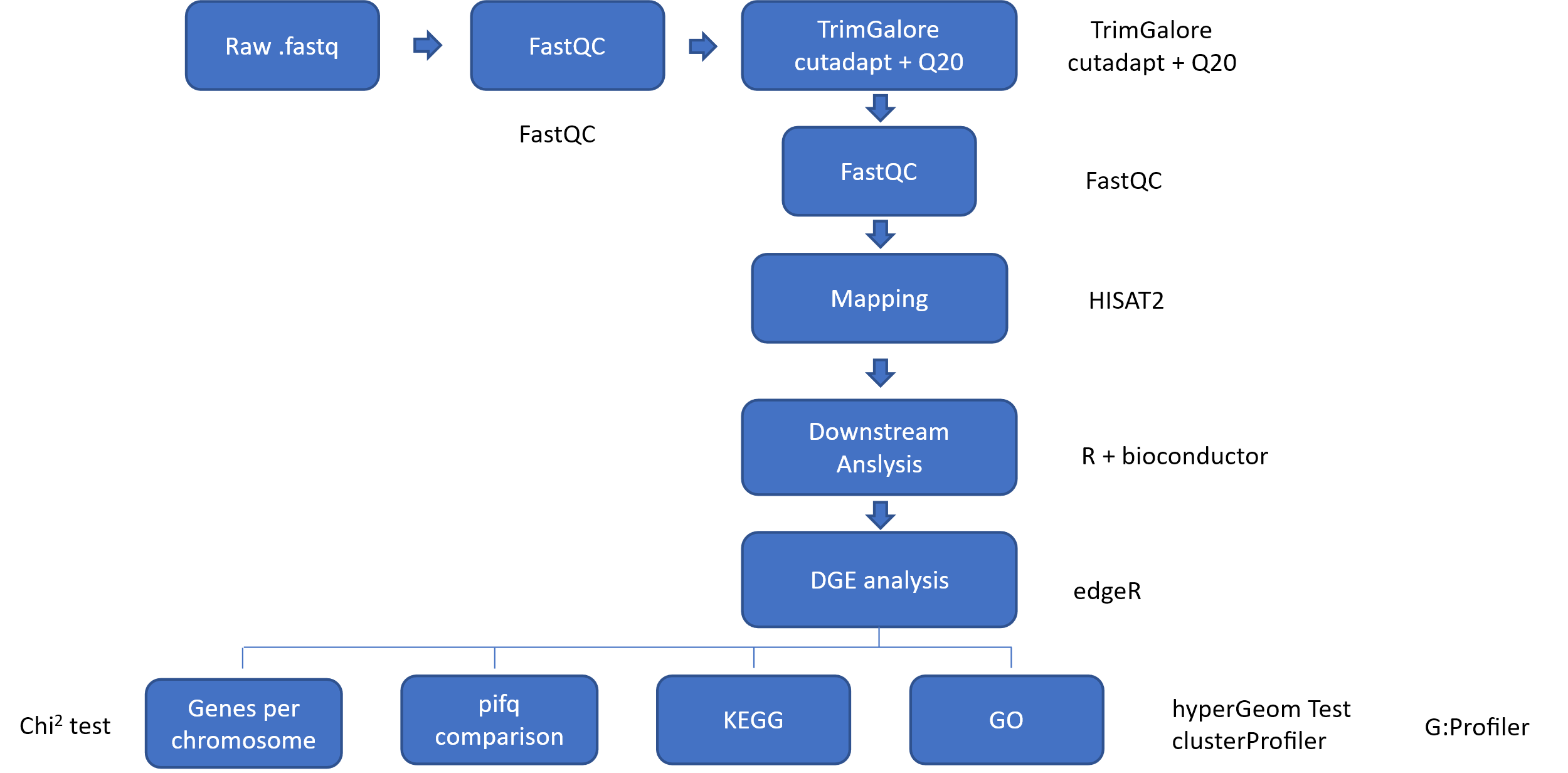


**Fig. S6** Data analysis pipeline of mRNAseq


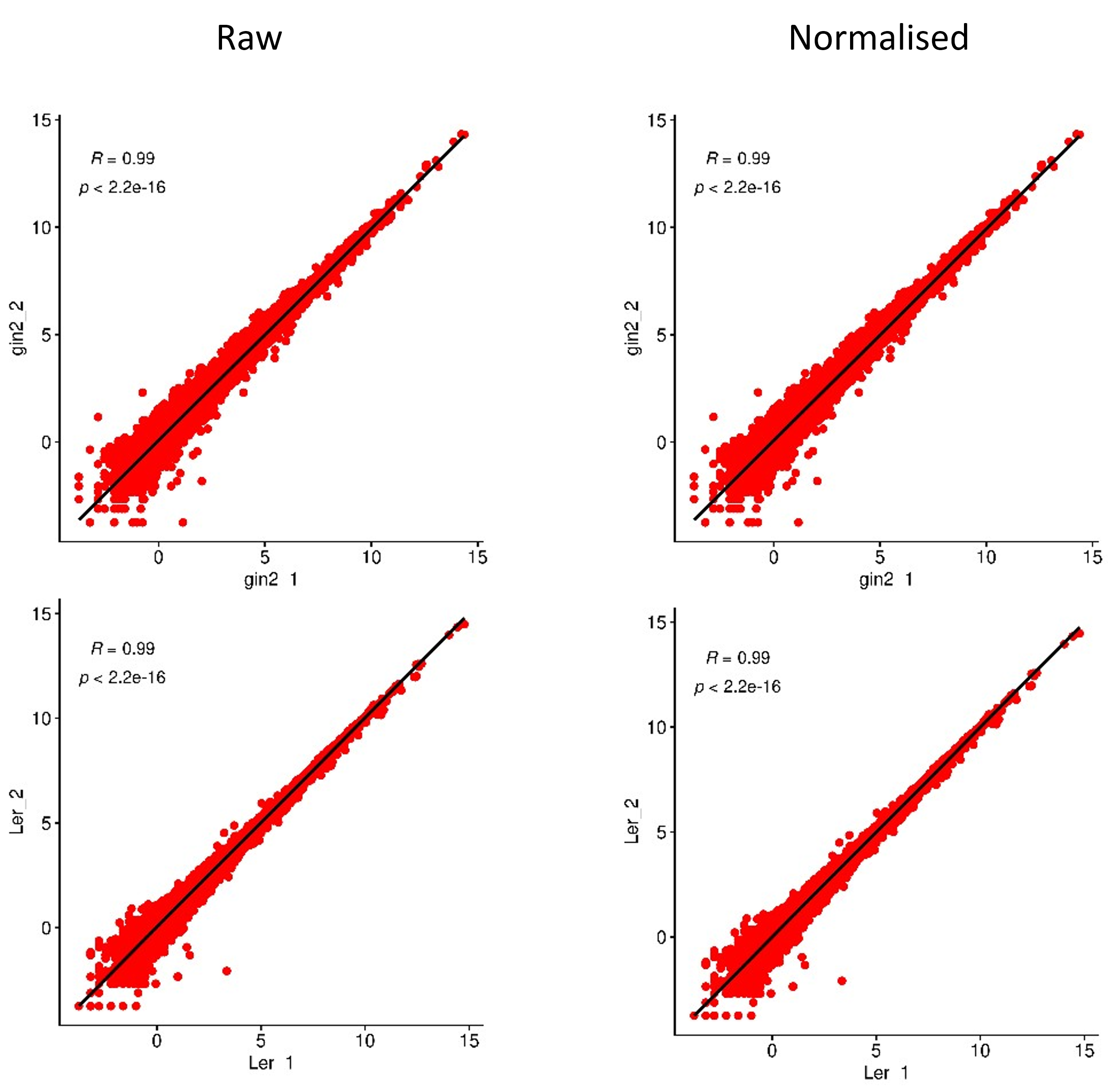


**Fig. S7** Person sample-similarity correlation of *gin2-1* and Ler samples used in mRNAseq

| **Category** | **Misregulated Genes** | **Total Expressed Genes** | **% Misregulation** |
| --- | --- | --- | --- |
| ***gin2-1* up** | **1,188** | **21,256** | **5.59** |
| ***gin2-1* down** | **1,156** | **21,256** | **5.44** |

**Table S1.** The proportions of mRNAseq *gin2-1* up/down genes (compared to Ler WT), represented in Fig. 3b.

(a)

**
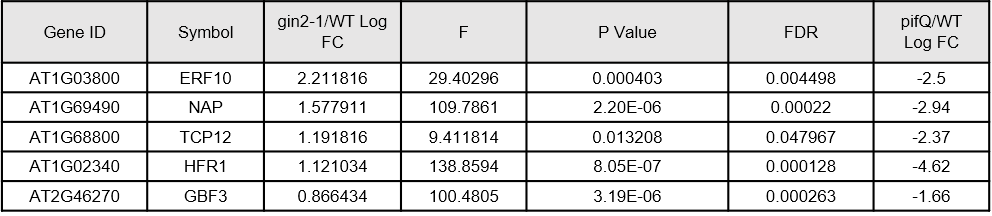
**

(b)

**
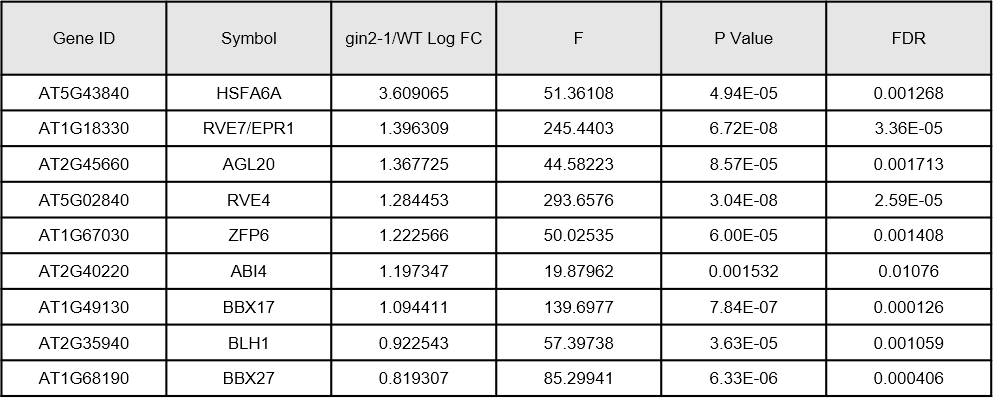
**

**Table S2.** (a) Selected light regulated genes from “*gin2-1* up, *pifQ* down” (a), and “*gin2-1* up only” (b) mRNAseq data.

**
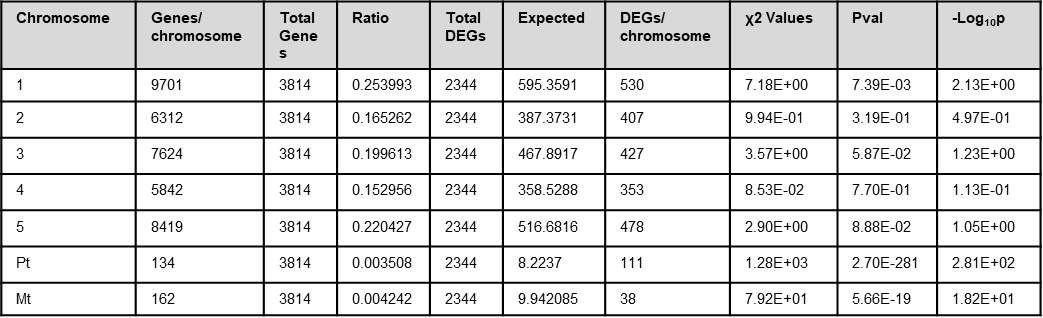
**

**Table S3.** Count tables at the gene level organised under chromosome and plastome. DEG=Differentially Expressed Genes.

| **Primer name** | **Sequence (5’-3’)** |
| --- | --- |
| **AG_2250_RPOA_F** | **GCGATGCGAAGAGCTTTACT** |
| **AG_2251_RPOA_R** | **CCAGGACCTTGGACACAAA** |
| **AG_2252_RPOB_F** | **GATGTGAGGTGGGTTCAGAA** |
| **AG_2253_RPOB_R** | **GGTCTCCCGTCTTGCAAATA** |
| **AG_2254_RPOC1_F** | **TTCTTCCTCCCGAGTTGAGA** |
| **AG_2255_RPOC1_R** | **CCACGGCTTCTTGTACCAAT** |
| **AG_2256_RPOC2_F** | **CGCGTCGACTTGTTGAAGTA** |
| **AG_2257_RPOC2_R** | **CGTCTGCTAAGACACGACCA** |
| **AG_2236_BCAT2_F** | **GGGATAATCTCGGGTTTGGT** |
| **AG_2237_BCAT2_R** | **CTTCATCCGGATAGCGTTGT** |
| **AG_2232_THDP_F** | **GACGAAGACGGACGAATCAT** |
| **AG_2233_THDP_R** | **TGCTGAAGCGATGTTAATGG** |
| **AG_2234_DIN2_F** | **CGGTCGTCGGAGAGAGTAAC** |
| **AG_2235_DIN2_R** | **GCCTTGCAAAACACCAAAAT** |
| **AG_2238_MCCA_F** | **CCCGTCTACAGGTCGAACAT** |
| **AG_2239_MCCA_R** | **ACCCGAACTGATGGTGAGAC** |
| **AG_2240_IVD_F_** | **ACTCTGTTGCGAGGGACTGT** |
| **AG_2241_IVD_R** | **CTTAGAAGGCGTCCTGTTGC** |
| **AG_2242_MCCB_F** | **CTTTGCCTTCAGGTGGGATA** |
| **AG_2243_MCCB_R** | **ACCGAGCAGCAATCTCTTGT** |
| **AG_1267_CAB2_F** | **CCCTGGAGACTACGGATG** |
| **AG_1267_CAB2_R** | **TCCAAACTTGACTCCGTTCC** |
| **AG_1428_CAA_F** | **TGAATACGCTGTCTTGCACC** |
| **AG_1429_CAA_R** | **TGTGATGGTGGTGGTAGCGA** |
| **DY_1166_HXK1_F** | **GGTTTCACTTTCTCGTTTCCTG** |
| **DY_1167_HXK1_R** | **CTTGTCCAACTGCTTCTTCG** |
| **AR027_PP2A_F** | **TAACGTGGCCAAAATGATGC** |
| **AR028_PP2A_R** | **GTTCTCCACAACCGCTTGGT** |
| **ERF10_F** | **GGAAGTGAGTGACAAGGGCGTA** |
| **ERF10_R** | **ATGGTCTCCTCCTTACACCCCT** |
| **AR077_HFR1_F** | **TGGCCATTACCACCGTTTAC** |
| **AR078_HFR1_R** | **AAACCGTGAAGAGACTGAGGAG** |
| **ZFP6_F** | **GAAGGGGACTTT ACGGAGGTG** |
| **ZFP6_R** | **GGTCCAGCCCA ATACCATTCT** |
| **ABI4_F** | **GAGATCCGAGAGCCACGTAA** |
| **ABI4_R** | **AGGGAGGAGAGGTCTTAGGG** |
| **SOC1_F** | **CGCAATTGTTCATTGGGTTA** |
| **SOC1_R** | **CAACAAGAGAGAAGCAGCTTTAGA** |

**Table S4.** Primers used in the course of this study
